# A Dynamic RCS and Noise Prediction and Reduction Method of Coaxial Tilt-Rotor Aircraft Based on Phase Modulation

**DOI:** 10.3390/s22249711

**Published:** 2022-12-11

**Authors:** Zeyang Wang, Jun Huang, Mingxu Yi, Shaoze Lu

**Affiliations:** School of Aeronautic Science and Engineering, Beihang University, Beijing 100083, China

**Keywords:** coaxial tilt-rotor aircraft, compass-scissors model, generated rotation matrix, carpet diagram, dynamic RCS, noise SPL

## Abstract

For tilt-rotor aircraft with coaxial rotors (coaxial rotor aircraft), reduction of radar cross section as well as acoustic noise can be essential for stealth design, and the rotation of the coaxial rotors can have an influence on noise and dynamic radar cross section (RCS) characteristics. In this paper, an approach to the prediction of both the sound pressure level (SPL) of noise and the dynamic RCS of coaxial-tilt aircraft is carried out, based on the theories of the FW-H equation, the physics optics method (PO) and the physical theory of diffraction (PTD) method. In order to deal with the rotating parts (mainly including coaxial rotors), a generated rotation matrix (GRM) is raised, aiming at giving a universal formula for the time-domain grid coordinate transformation of all kinds of rotation parts with arbitrary rotation centers and rotation axis directions. Moreover, a compass-scissors model (CSM) reflecting the phase characteristics of coaxial rotors is established, and a method of noise reduction and RCS reduction based on the phase modulation method is put forward in this paper. The simulation results show that with proper CSM parameter combinations, the reduction of noise SPL can reach approximately 3~15 dB and the reduction of dynamic RCS can reach 1.6 dBsm at most. The dynamic RCS and noise prediction and reduction method can be meaningful for the radar-acoustic stealth design of coaxial tilt-rotor aircrafts.

## 1. Introduction

With the abilities of vertical take-off and landing, hovering and high-speed cruise flight, tilt-rotor aircraft can be regarded as a combination of helicopters and fixed-wing aircraft, which makes it have a broad prospect of application in military and civil aviation [1,2,3]. The CL-84 Dynavert and V-22 “Osprey” are well known as successful examples of tilt-rotor aircraft. With the development of artificial intelligence and autonomous technology, tilt-rotor aircraft are widely used in the field of unmanned aerial vehicles (UAVs) for photography tasks, recon missions, etc. Cetinsoy et al. have designed and constructed a new quad tilt-wing UAV [4]. Franchi et al. have developed a tilt-hexarotor UAV [5]. 

The typical construction of rotor system of tilt-rotor aircraft usually consists of single rotors, and the rotation speeds of rotors are limited by the requirement of torque balance. Coaxial rotors can solve this problem, as the torque of an independent coaxial rotor can always be 0 when the two rotors rotate at the same speed in opposite directions. Therefore, coaxial rotor aircraft have the advantage of free adjustment of rotation speeds without producing extra torques. Aircraft with coaxial rotors have broad application prospects, and research on coaxial rotors has become a hotspot in recent years [6,7,8,9]. Lv et al. have designed and assembled a coaxial tilt-rotor unmanned aerial vehicle (CTRUAV) with a novel structure and possesses two pairs of front coaxial tiltable rotors that have the advantages of balanced Coriolis force, balanced reaction torques and balanced torques caused by blade flapping on the coaxial rotors [10]. 

As for the military coaxial tilt-rotor for recon missions, acoustic noise and radar cross section (RCS) can be significant characteristic signals that can make the coaxial tilt-rotor easily detected by enemy radars and noise detectors. For these reasons, research on the noise and RCS characteristics and the noise and RCS reduction method is necessary to the survival ability of coaxial tilt-rotor aircraft.

For a few decades, the aerodynamic shape design of the blades has been the main approach to the reduction of noise generated by aircraft rotors [11,12,13], with the main design parameters including airfoil, chord length, twist distribution, blade radius, tip sweepback angle, etc. These passive noise reduction methods have to change the aerodynamic characteristics of the rotor and might have negative effects on the helicopter’s rotor. The passive noise reduction design above, based on the change in the rotor’s aerodynamic shape, can sometimes conflict with the dynamic design, and the effects of noise cancellation have to be limited. Therefore, active noise cancellation (ANC) methods have also been developed in recent years. The high-harmonic control (HHC) method is regarded as one of the typical ANC methods [14]. Ma et al. have proposed an active rotor control technology based on higher harmonic control (HHC), aiming at the reduction of noise produced by tilt-rotor aircraft [15]. Taking the periodicity of the rotation of rotors into account, another sort of ANC method based on the phase modulation method has been proposed. As for the rotor system with multiple rotors rotating at the same rotation speed, each of the rotors can be regarded as an individual sound source, and the noise can be modified by only adjusting the initial angles of the rotors. Moreover, the shapes of the blades are not changed, and the noise cancellation can be realized without changing the of dynamic characteristics [16,17,18,19,20,21,22,23,24]. Guan et al. have carried out a noise attenuation experiment with a quadrotor using the phase synchronization method [25]. Ma et al. have developed an aeroacoustics suppression method for tilt-rotor aircraft based on rotor phase control [26]. 

Advanced radar systems can also be a serious threat to rotor aircraft, especially radar systems with joint detection strategies [27]. Therefore, a reduction of RCS is essential for the stealth design of rotor aircraft. As for the RCS prediction method of rotor aircraft, the Physics Optics method (PO) and Physical Theory of Diffraction (PTD) method have always been effective methods that are widely used. Based on the PO and PTD methods, Zhou ZY et al. have developed a dynamic scattering method (DSM) that can give predictions for aircraft models with rotating parts [28,29,30]. DSM is effective for the calculation of the dynamic RCS of rotor aircraft, using the grid coordinate transformation matrixes applied in DSM. The research on dynamic RCS calculation carried out currently contains all kinds of models, such as ordinary helicopters with a single or coaxial rotor, aircraft with ducted tail rotor and tilt-rotor aircraft. However, little research has operated using the dynamic method for noise calculation. Moreover, the grid coordinate transformation matrixes in DSM mainly deal with rotating elements with the rotation axis parallel to the axis of coordinates *(xyz*). 

Generally speaking, most of the previous research concentrated on the prediction of acoustic noise and RCS of rotor aircraft and the unilateral reduction of acoustic noise or unilateral reduction of RCS. However, facing multiple threats from radars, noise detectors, etc., unilateral reduction of acoustic noise or unilateral reduction of RCS are not enough for the stealth of the coaxial tilt-rotor aircraft, and a method of both noise reduction and RCS reduction is required for the survival ability of coaxial tilt-rotor aircraft. Moreover, little previous research has been put forward on the stealth design of coaxial tilt-rotor aircraft. In this paper, a dynamic RCS and noise prediction and reduction method for coaxial tilt-rotor aircraft is proposed based on the theory of the FW-H equation, theory of the PO method and phase modulation method. The noise SPL values and the dynamic RCS values of coaxial tilt-rotor aircraft at different phase situations are analyzed in this paper, and the optimal phase parameter combinations are selected for the stealth design of coaxial tilt-rotor aircraft. 

## 2. Theories and Methodologies

### 2.1. FW-H Equation for Noise Prediction

The application of the noise prediction method is based on the FW-H equation, which has been developed by Ffowcs Williams and Hawkings according to the Lighthill acoustic analogy method. The solution formula for the FW-H equation in the time domain has been put forward by Farassat as Farassat formulation 1A.

(1) $$p^{\prime }={p^{\prime }}_{T}+{p^{\prime }}_{L}$$

F.Farassat has developed a direct calculation method of the sound field of rotating sound sources based on the FW–H equation and also developed several time-domain formulations for the solution of the FW–H equation [31]. Moreover, the Kirchhoff equation for the calculation of the sound field has been deduced, which can be written as:

(2) $$4\pi {p^{\prime }}_{T}\left(x,t\right)={\int_{S}\left[\frac{\rho_{0}{\dot{v}}_{n}}{r{\left(1-M_{r}\right)}^{2}}\right]}_{ret}dS+{\int_{S}\left[\frac{\rho_{0}v_{n}\left(r{\dot{M}}_{i}{\hat{r}}_{i}+c_{0}M_{r}-c_{0}M^{2}\right)}{r^{2}{\left(1-M_{r}\right)}^{3}}\right]}_{ret}dS$$

(3) $$\begin{array}{l} 4\pi {p^{\prime }}_{L}\left(x,t\right)=\frac{1}{c_{0}}{\int_{S}\left[\frac{{\dot{l}}_{i}{\hat{r}}_{i}}{r{\left(1-M_{r}\right)}^{2}}\right]}_{ret}dS+{\int_{S}\left[\frac{l_{r}-l_{i}M_{i}}{r^{2}{\left(1-M_{r}\right)}^{2}}\right]}_{ret}dS\\ +\frac{1}{c_{0}}{\int_{S}\left[\frac{l_{r}\left(r{\dot{M}}_{i}{\hat{r}}_{i}+c_{0}M_{r}-c_{0}M^{2}\right)}{r^{2}{\left(1-M_{r}\right)}^{3}}\right]}_{ret}dS \end{array}$$

where $p^{\prime }$ represents the total sound pressure, ${p^{\prime }}_{T}$ is the thickness noise pressure and ${p^{\prime }}_{L}$ is the loading noise pressure. $c_{0}$ is the sound speed and the subscript “ret” means the sound pressure value calculated at retarded time. 

According to finite element method (FEM), the geometry model of the rotor blades can be divided into triangle facet elements for the integral calculations of thickness and loading noise. Therefore, Equations (2) and (3) can be transformed into:

(4) $${p^{\prime }}_{T}\left(x,t\right)=\frac{1}{4\pi }\sum_{i=1}^{N}\left(\frac{\rho_{0}{\dot{v}}_{n}}{r{\left(1-M_{r}\right)}^{2}}\Delta S_{i}+\frac{\rho_{0}v_{n}\left(r{\dot{M}}_{i}{\hat{r}}_{i}+c_{0}M_{r}-c_{0}M^{2}\right)}{r^{2}{\left(1-M_{r}\right)}^{3}}\Delta S_{i}\right)$$

(5) $${p^{\prime }}_{L}\left(x,t\right)=\frac{1}{4\pi }\sum_{i=1}^{N}\left(\frac{1}{c_{0}}\frac{{\dot{l}}_{i}{\hat{r}}_{i}}{r{\left(1-M_{r}\right)}^{2}}\Delta S_{i}+\frac{l_{r}-l_{i}M_{i}}{r^{2}{\left(1-M_{r}\right)}^{2}}\Delta S_{i}+\frac{1}{c_{0}}\frac{l_{r}\left(r{\dot{M}}_{i}{\hat{r}}_{i}+c_{0}M_{r}-c_{0}M^{2}\right)}{r^{2}{\left(1-M_{r}\right)}^{3}}\Delta S_{i}\right)$$

In Equations (4) and (5), *r_i_* is determined by the position of facet element numbered *i*, *M_i_* is determined by the position as well as the rotation speed of facet element numbered *i*, and thus the thickness and loading noise of each facet element can both be regarded as functions of its position and rotation speed. Therefore, Equations (4) and (5) can be written as:

(6) $${p^{\prime }}_{T}\left(x,t\right)=\frac{1}{4\pi }\sum_{i=1}^{N}\left(THICK\left(\omega_{i},X\left(\Delta S_{i}\right)\right)\right)$$

(7) $${p^{\prime }}_{L}\left(x,t\right)=\frac{1}{4\pi }\sum_{i=1}^{N}\left(LOAD\left(\omega_{i},X\left(\Delta S_{i}\right)\right)\right)$$

In the hovering status and the low-speed steady flight, the aerodynamic noise mainly consists of the thickness noise and the loading noise, and therefore the Farassat formulation 1A can give a proper prediction for the noise generated by the coaxial rotors in the hovering status and the low-speed steady flight. The total noise pressure equals the linear superimposition of the thickness noise and loading noise produced by each of the rotors.

### 2.2. PO and PTD Method for RCS Calculation

**Figure 1:**
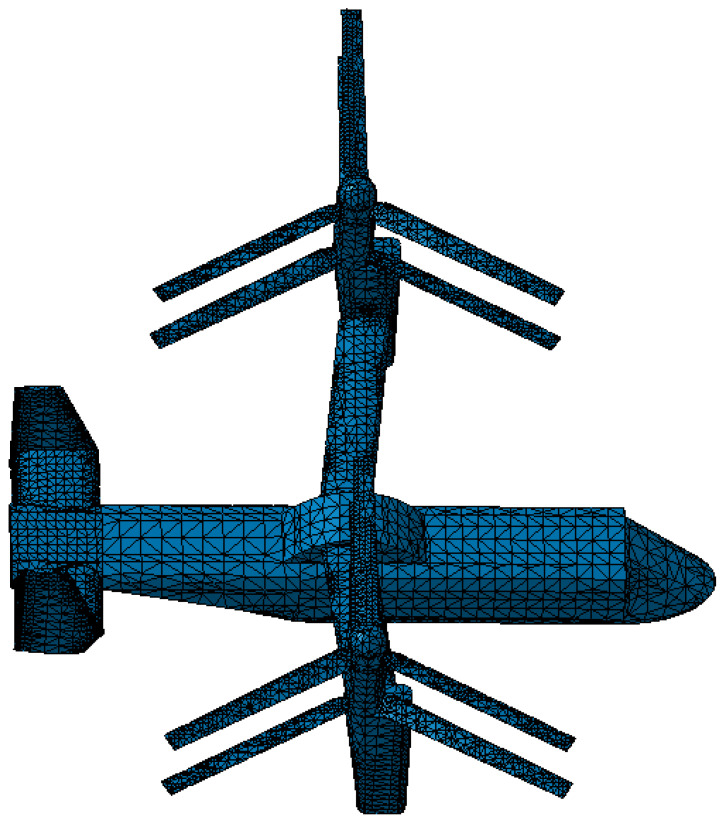
Aircraft geometry model divided into triangle elements.

As an effective method of RCS prediction, the PO method is applied to the prediction of RCS for coaxial tilt-rotor aircraft. According to the finite element method (FEM), the geometry model of the target can be divided into triangle elements, as shown in Figure 1, and the sizes of mesh generations for different parts of the aircraft model are listed in Table 1. The PO method can be applied to the calculation of all triangular facets. 

The far-field electric field can be obtained by:

(8) $$E_{S}=\frac{e^{-jkr}}{4\pi r}\left(-j\omega \mu \right)\int_{S}J_{S}e^{jk\hat{s}\cdot r’}dS+jk\hat{s}\frac{1}{\varepsilon }\int_{S}\rho_{S}e^{jk\hat{s}\cdot r’}dS$$

where $E_{S}$ represents the scattered electric field, *r* represents the field point, r’ represents the coordinate vector of source point. $J_{S}$ represents the surface current, which can be calculated by:

(9) $$J_{S}=2\hat{n}\times H_{i}$$

In Equation (9), $H_{i}$ represents the magnet field of the incident wave. 

For far field, the incoming wave can be considered a plain wave, and thus the RCS of the target can be written as:

(10) $$\sqrt{\sigma }=\sqrt{4\pi r^{2}}\frac{1}{\left|E_{i}\right|}\left(-j\omega \mu \frac{e^{-jkr}}{4\pi r}\int_{S}\hat{e_{s}}\cdot J_{S}e^{jk\hat{s}\cdot r’}dS\right)$$

Substitute Equation (8) into Equation (10), it can be obtained that

(11) $$\sqrt{\sigma }=\sqrt{4\pi r^{2}}\frac{1}{\left|E_{i}\right|}\left(-j\omega \mu \frac{e^{-jkr}}{4\pi r}\cdot 2\int_{S}\hat{e_{s}}\cdot \left(\hat{n}\times H_{i}\right)e^{jk\hat{s}\cdot r’}dS\right)$$

Taking the relationship between the magnetic field and the electronic field, the calculation of RCS can be transferred into:

(12) $$\sqrt{\sigma }=\frac{jk}{\sqrt{\pi }}\hat{n}\cdot \left({\hat{e}}_{s}\times {\hat{h}}_{i}\right)e^{jkw\cdot r_{0}}I^{\prime }$$

(13) w=s−i

In Equation (12), the integral item $I^{\prime }$ can be written as:

(14) $$I^{\prime }=\left\{ \begin{array}{l} \frac{e^{jkw\cdot r_{m}}}{jk{\left|p\right|}^{2}}\sum_{m=1}^{N}p\cdot L_{m}\mathrm{sinc}\left(\frac{kw\cdot L_{m}}{2}\right),\left|p\right|\neq 0\\ A,\left|p\right|=0 \end{array}\right.$$

where *N* represents the number of sides of every mesh element. *N =* 3 for triangle mesh elements, which are most commonly used in mesh generation. 

In order to obtain more accurate RCS results, the edge diffraction effects of the target should also be taken into consideration, and PTD is applied to the RCS calculation of the target. More details of PTD can be seen in References [28,29,30], and the total RCS can be obtained by:

(15) $$\sigma \left(t\right)={\left|\sum_{i=1}^{N_{F}\left(t\right)}{\left(\sqrt{\sigma_{F}\left(t\right)}\right)}_{i}+\sum_{j=1}^{N_{E}\left(t\right)}{\left(\sqrt{\sigma_{E}\left(t\right)}\right)}_{i}\right|}^{2}$$

where elements with subscript *E* represent the edge elements and elements with subscript *F* represent the face elements. 

Moreover, the essence of the time-variation of dynamic RCS can be explained as the time-variation of position of facet and edge elements, and RCS is determined by the relative positions of the elements and the observer point. Thus, the total dynamic RCS can be regarded as a function of the position of each element. 

### 2.3. Dynamic Calculation Formulas with Generalized Rotation Matrix (GRM)

As for targets with moving parts, such as helicopters with rotating rotors, the noise and RCS values of these sorts of targets change over time. It is essential that a dynamic method be introduced for the time-domain solution of these targets. Zhou ZY has developed a method called the dynamic scattering method (DSM), which can be applied to dealing with the RCS of rotating rotors. However, the grid coordinate transformation matrixes of the DSM seem to be separated into different formulas, which leads to difficulties in comprehension and concise expressions for rotation model establishment. Moreover, the expressions of the position of the rotation center and the direction of the rotation axis in the grid coordinate transformation matrixes of DSM are rather vague. Therefore, based on the grid coordinate transformation matrixes of DSM, a concise and comprehensive matrix called generalized rotation matrix (GRM) is put forward, aiming at giving a universal formula for the time-domain grid coordinate transformation of all kinds of rotation parts with arbitrary rotation centers and rotation axis directions. 

**Figure 2:**
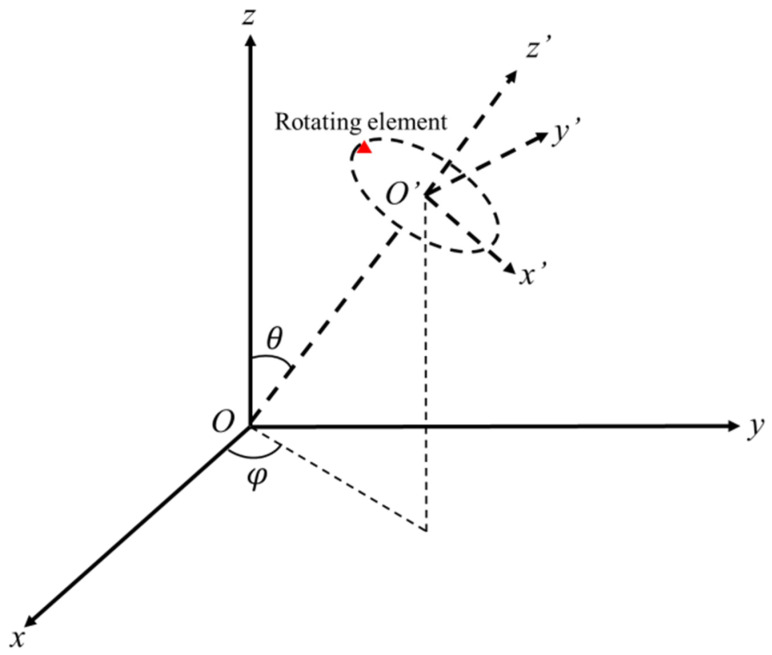
Schematic diagram of rotating element and rotation axis.

As is shown in Figure 2, the situation of an element rotating around one rotation axis is taken into consideration. The coordinate of rotation center is set as $X_{0}={\left[{\begin{array}{ccc} x_{0} & y_{0} & z \end{array}}_{0}\right]}^{T}$, and the direction vector of the rotation axis is set as $n={\left[\cos\varphi \sin\theta ,\sin\varphi \sin\theta ,\cos\theta \right]}^{T}$. Set $O^{\prime }x^{\prime }y^{\prime }z^{\prime }$ as the coordinate system, with $z^{\prime }$ axis parallel to the rotation axis and $z^{\prime }O^{\prime }x^{\prime }$ plane vertical to xOy plane. The position vector of the rotating element in $O^{\prime }x^{\prime }y^{\prime }z^{\prime }$ is set as ${\left[\begin{array}{ccc} x^{\prime } & y^{\prime } & z^{\prime } \end{array}\right]}^{T}$, and $O^{\prime }$ is considered as the rotation center, whose position vector is ${\left[{\begin{array}{ccc} x_{0} & y_{0} & z \end{array}}_{0}\right]}^{T}$.

The transformation relationship between the coordinates Oxyz and $O^{\prime }x^{\prime }y^{\prime }z^{\prime }$ can be obtained as:

(16) $$\left[\begin{array}{l} x-x_{0}\\ y-y_{0}\\ z-z_{0} \end{array}\right]=\left[\begin{array}{ccc} \cos\varphi & -\sin\varphi & 0\\ \sin\varphi & \cos\varphi & 0\\ 0 & 0 & 1 \end{array}\right]\left[\begin{array}{ccc} \cos\theta & 0 & -\sin\theta \\ 0 & 1 & 0\\ \sin\theta & 0 & \cos\theta \end{array}\right]\left[\begin{array}{l} x^{\prime }\\ y^{\prime }\\ z^{\prime } \end{array}\right]$$

When the element starts to rotate around the rotation axis with the angular velocity of ω, ${\left[\begin{array}{ccc} x^{\prime } & y^{\prime } & z^{\prime } \end{array}\right]}^{T}$ and the initial position vector ${\left[\begin{array}{ccc} x^{\prime } & y^{\prime } & z^{\prime } \end{array}\right]}_{t=0}^{T}$ have the transformation relationship of:

(17) $$\left[\begin{array}{l} x^{\prime }\\ y^{\prime }\\ z^{\prime } \end{array}\right]=\left[\begin{array}{ccc} \cos\left(\omega t\right) & -\sin\left(\omega t\right) & 0\\ \sin\left(\omega t\right) & \cos\left(\omega t\right) & 0\\ 0 & 0 & 1 \end{array}\right]{\left[\begin{array}{l} x^{\prime }\\ y^{\prime }\\ z^{\prime } \end{array}\right]}_{t=0}$$

Therefore, the relationship between the absolute position vector ${\left[\begin{array}{ccc} x & y & z \end{array}\right]}^{T}$ and the initial $O^{\prime }x^{\prime }y^{\prime }z^{\prime }$ position vector ${\left[\begin{array}{ccc} x^{\prime } & y^{\prime } & z^{\prime } \end{array}\right]}_{t=0}^{T}$ can be written as:

(18) $$\left[\begin{array}{l} x\left(t\right)\\ y\left(t\right)\\ z\left(t\right) \end{array}\right]=\left[\begin{array}{ccc} \cos\varphi & -\sin\varphi & 0\\ \sin\varphi & \cos\varphi & 0\\ 0 & 0 & 1 \end{array}\right]\left[\begin{array}{ccc} \cos\theta & 0 & -\sin\theta \\ 0 & 1 & 0\\ \sin\theta & 0 & \cos\theta \end{array}\right]\left[\begin{array}{ccc} \cos\left(\omega t\right) & -\sin\left(\omega t\right) & 0\\ \sin\left(\omega t\right) & \cos\left(\omega t\right) & 0\\ 0 & 0 & 1 \end{array}\right]{\left[\begin{array}{l} x^{\prime }\\ y^{\prime }\\ z^{\prime } \end{array}\right]}_{t=0}+\left[\begin{array}{l} x_{0}\\ y_{0}\\ z_{0} \end{array}\right]$$

At the time point of *t* = 0, the absolute position vector and the initial $O^{\prime }x^{\prime }y^{\prime }z^{\prime }$ position vector also satisfy the relationship of Equation (18), as is illustrated in Equation (19):

(19) $$\left[\begin{array}{l} x\left(t=0\right)-x_{0}\\ y\left(t=0\right)-y_{0}\\ z\left(t=0\right)-z_{0} \end{array}\right]=\left[\begin{array}{ccc} \cos\varphi & -\sin\varphi & 0\\ \sin\varphi & \cos\varphi & 0\\ 0 & 0 & 1 \end{array}\right]\left[\begin{array}{ccc} \cos\theta & 0 & -\sin\theta \\ 0 & 1 & 0\\ \sin\theta & 0 & \cos\theta \end{array}\right]\left[\begin{array}{l} x^{\prime }\\ y^{\prime }\\ z^{\prime } \end{array}\right]$$

Combine Equation (18) with Equation (19), it can be obtained that the absolute position of the rotation element can be considered as a function of time, which can be written as:

(20) $$X\left(t\right)=R_{\varphi }\left(\varphi \right)R_{\theta }\left(\theta \right)R_{\omega }\left(\omega ,t\right){\left(R_{\theta }\left(\theta \right)\right)}^{-1}{\left(R_{\varphi }\left(\varphi \right)\right)}^{-1}\left(X\left(t=0\right)-X_{0}\right)+X_{0}$$

In Equation (20), the generated rotation matrix (GRM) can be obtained as:

(21) $$R_{GRM}\left(\omega ,t\right)=R_{\varphi }\left(\varphi \right)R_{\theta }\left(\theta \right)R_{\omega }\left(\omega ,t\right){\left(R_{\theta }\left(\theta \right)\right)}^{-1}{\left(R_{\varphi }\left(\varphi \right)\right)}^{-1}$$

where

(22) $$R_{\varphi }\left(\varphi \right)=\left[\begin{array}{ccc} \cos\varphi & -\sin\varphi & 0\\ \sin\varphi & \cos\varphi & 0\\ 0 & 0 & 1 \end{array}\right]$$

(23) $$R_{\theta }\left(\theta \right)=\left[\begin{array}{ccc} \cos\theta & 0 & -\sin\theta \\ 0 & 1 & 0\\ \sin\theta & 0 & \cos\theta \end{array}\right]$$

(24) $$R_{\omega }\left(\omega ,t\right)=\left[\begin{array}{ccc} \cos\left(\omega t\right) & -\sin\left(\omega t\right) & 0\\ \sin\left(\omega t\right) & \cos\left(\omega t\right) & 0\\ 0 & 0 & 1 \end{array}\right]$$

(25) $$X\left(t\right)={\left[\begin{array}{ccc} x\left(t\right) & y\left(t\right) & z\left(t\right) \end{array}\right]}^{T}$$

(26) $$X_{0}={\left[{\begin{array}{ccc} x_{0} & y_{0} & z \end{array}}_{0}\right]}^{T}$$

From Equation (21), it can be seen that GRM contains the information of time, rotation speed and direction of the rotation axis. The rotation speed equals to 0 when the rotation element keeps still, and then

(27) $$\left\{ \begin{array}{l} R_{GRM}\left(\omega ,t\right)\equiv E\\ X\left(t\right)=X\left(t=0\right) \end{array}\right.$$

indicating the stationary state of the rotation element. 

The whole model of coaxial tilt-rotor aircraft can be considered as consisting of rotating parts (rotors) and steady parts (fuselages), which can be written as:

(28) $$M\left(t\right)=\left[P_{rotor1}\left(t\right),P_{rotor2}\left(t\right),P_{rotor3}\left(t\right),P_{rotor4}\left(t\right),P_{fuselage}\left(t\right)\right]$$

According to the finite element method (FEM), the parts can be written as matrix of Equation (29):

(29) $$P\left(t\right)={\left[X_{1}\left(t\right),X_{2}\left(t\right),\ldots ,X_{n}\left(t\right)\right]}_{3\times n}$$

The acoustic pressure of noise can be obtained by:

(30) $$\begin{array}{l} p\left(M\left(t\right)\right)=\frac{1}{4\pi }\sum_{i=1}^{N}\left(THICK\left(\omega_{i},\left(R_{GRM}\left(\omega_{i},t\right)\left(X_{rotor,i}\left(t=0\right)-X_{0}\right)+X_{0}\right)\right)\right)\\ +\frac{1}{4\pi }\sum_{i=1}^{N}\left(LOAD\left(\omega_{i},\left(R_{GRM}\left(\omega_{i},t\right)\left(X_{rotor,i}\left(t=0\right)-X_{0}\right)+X_{0}\right)\right)\right) \end{array}$$

For the calculation of dynamic RCS, the model matrix can be written as:

(31) $$M\left(t\right)=\left[P_{Fm}\left(t\right),P_{Em}\left(t\right),P_{Fs}\left(t\right),P_{Es}\left(t\right)\right]$$

where $P_{Fm}\left(t\right)$ represents the set of moving facet elements, $P_{Em}\left(t\right)$ represents the set of moving edge elements, $P_{Fs}\left(t\right)$ represents the set of static facet elements and $P_{Es}\left(t\right)$ represents the set of static edge elements. 

Therefore, the dynamic RCS can be obtained by:

(32) $$\sigma \left(M\left(t\right)\right)={\left|FM\left(t\right)+EM\left(t\right)+FS+ES\right|}^{2}$$

where

(33) $$\left\{ \begin{array}{l} FM\left(t\right)=\sum_{i=1}^{N_{Fm}}\left(\sqrt{\sigma_{F}\left(R_{GRM}\left(\omega_{i},t\right)\left(X_{Fm,i}\left(t=0\right)-X_{0,i}\right)+X_{0,i}\right)}\right)\\ EM\left(t\right)=\sum_{j=1}^{N_{Em}}\left(\sqrt{\sigma_{E}\left(R_{GRM}\left(\omega_{j},t\right)\left(X_{Fm,j}\left(t=0\right)-X_{0,j}\right)+X_{0,j}\right)}\right)\\ FS=\sum_{k=1}^{N_{Fs}}\left(\sqrt{\sigma_{F}\left(X_{Fs,k}\right)}\right)\\ ES=\sum_{m=1}^{N_{Es}}\left(\sqrt{\sigma_{E}\left(X_{Es,m}\right)}\right) \end{array}\right.$$

### 2.4. Verifications

#### 2.4.1. Noise Prediction

For the verification of the noise prediction method put forward above, the experimental data [32] of the noise of a sort of rotor is used. The rotor is made up of two blades that are equally placed, with the airfoil of NACA0010, a chord of 0.4 m and 10 m in diameter. The speed of sound is *c_0_* = 340.75 m/s and the air density is $\rho_{0}$ = 1.234 kg/m^3^. The rotor runs in the case where the inflow Mach number is 0.2, parallel to the rotation axis. The inflow Mach vector can be obtained as (0, 0, 0.2). The blade tip Mach is 0.6. The distance between the observer and the rotation axis is 50 m, and the location of the observer is in the XY plane, the rotation plane. 

**Figure 3:**
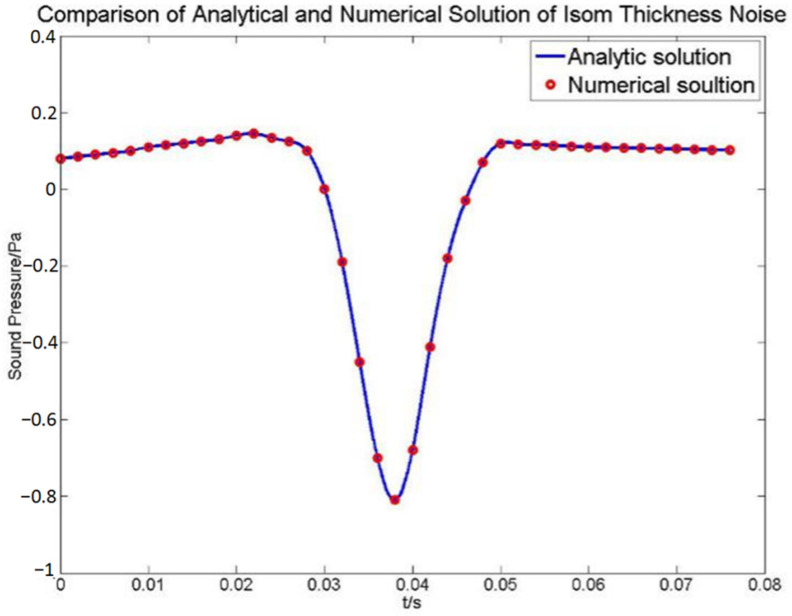
Comparison of sound pressure between the calculated result and experimental data of an experimental rotor.

The comparison of the sound pressure of the experimental results and the results calculated using the method is shown in Figure 3. It can be clearly illustrated from Figure 3 that the calculated results duplicate the experimental results quite well, which can prove the validity of the method for calculating acoustic noise. 

#### 2.4.2. Dynamic RCS Prediction

The experimental data of the dynamic RCS of a sort of tilt-rotor aircraft model [28] is applied for the verification of the dynamic RCS prediction method put forward above. The blade passing period of the rotor has been divided into time points, and different geometry models at different time points have participated in the calculation in FEKO. The dynamic RCS is calculated using the method developed above. The radar wave frequency is 5 GHz, and the rotation speed is fixed at 1600 r/min.

**Figure 4:**
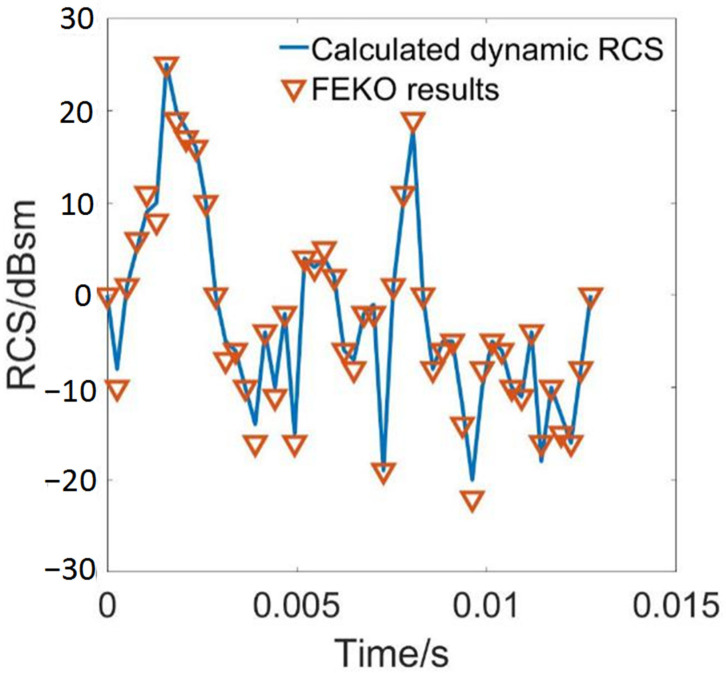
Comparison of dynamic RCS between the calculated result and FEKO result of a tilt-rotor model.

The comparison of the dynamic RCS curve of the experimental results and the dynamic RCS curve calculated using the method is shown in Figure 4, indicating that the calculated results fit the FEKO results quite well, which can prove the validity of the method for dynamic RCS calculation.

## 3. Models for Simulations

### 3.1. Geometry Model of Coaxial Tilt-Rotor Aircraft

**Figure 5:**
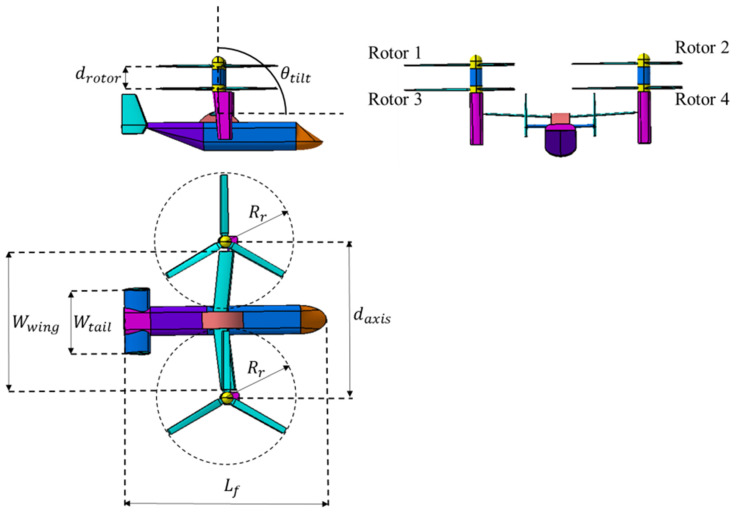
Three views of geometry model of coaxial tilt-rotor aircraft.

The geometry model of coaxial tilt-rotor aircraft is established according to the geometry parameters of V22 tilt-rotor aircraft, as is shown in Figure 5. $R_{r}$ represents the radius of the rotors, and $d_{rotor}$ represents the distance between the lower rotor and upper rotor of each coaxial rotor. $d_{axis}$ is the distance between the rotation axis of the two coaxial rotors. As for the fuselage, $L_{f}$ represents the length of the fuselage, $W_{wing}$ represents the wingspan of the main wings and $W_{tail}$ represents the wingspan of the horizontal tail. The numerical values for the geometry parameters are listed in Table 2.

### 3.2. Compass-Scissors Model (CSM) of Coaxial Rotors

With the two rotors rotating at the same rotation speed in opposite directions, a coaxial rotor appears to have obvious symmetry and directivity. A model called the compass-scissors model (CSM) has been put forward for the description of the orientation and rotation phase status of coaxial rotors. The descriptions of the main elements and the main parameters of CSM are as follows.

a. CCW rotor, CW rotor and pointer blades

**Figure 6:**
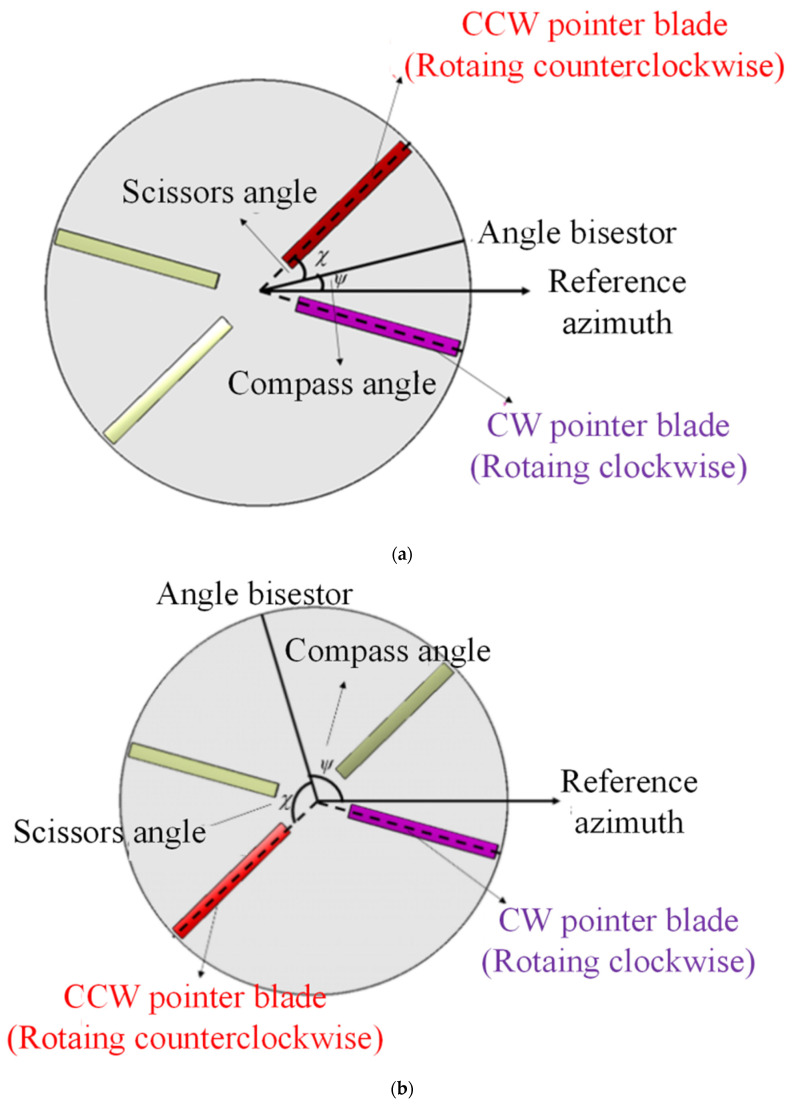
Schematic diagram and model elements of CSM for coaxial rotor: (**a**) Compass angle ≤ 90°, (**b**) Compass angle > 90°.

A coaxial rotor consists of two rotors rotating in opposite directions. The rotor rotating counterclockwise is defined as a CCW rotor, while the rotor rotating clockwise is defined as a CW rotor. For each rotor of the coaxial rotor, one of the blades can be selected to be the pointer blade, and the angle formed by the pointer blade and the defined reference direction can be regarded as the direction angle of the rotor. The elements mentioned above are shown in Figure 6.

b. Compass angle and scissors angle

Despite the rotation of the CCW rotor and the CW rotor (the rotor rotating clockwise), the angle bisector of the pointer blades of the two rotors remains still, just like the pointer of a compass. The angle formed by the angle bisector and the defined reference direction is defined as “compass angle”, determining the orientation status of the coaxial rotor. The signal for the compass angle is set as ψ in this paper.

With the passing of time, the pointer blades rotating away from the angle bisector can form an “opening scissors”. Thus, the angle formed by the angle bisector and one of the pointer blades can be defined as the “scissors angle”, reflecting the rotation phase status of the coaxial rotor. Therefore, the model can be named as a compass-scissors model (CSM). The signal of the scissors angle is set as χ in this paper.

By observing the main elements and the main parameters of CSM, it can be inferred that CSM has the ability to describe the orientation-time status of coaxial rotor. The orientation status of a coaxial rotor is determined by compass angle, while the time status of a coaxial rotor is determined by the scissors angle. Obviously, coaxial rotors with the same rotation axis and rotation speed but different compass angles and different scissors angles can lead to quite different noise and RCS at the same observer point.

**Figure 7:**
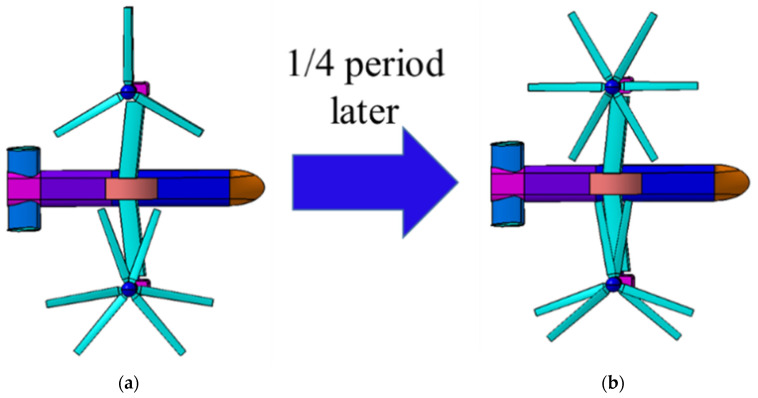
The relationship between situation “left scissors angle = 0°, right scissors angle = 20°” (**a**) and situation “left scissors angle = 90°, right scissors angle = 110°” (**b**).

As for coaxial tilt-rotor aircraft, the rotor system consists of two coaxial rotors. According to the periodicity of coaxial rotors, different scissors angles of two coaxial rotors can illustrate the same situation as long as the scissors angle difference of the two coaxial rotors stays the same. For example, the situation of “left scissors angle = 90°, right scissors angle = 110°” represents the situation of “left scissors angle = 0°, right scissors angle = 20°” with time passing *T*/4 (T=2π/ω), as is shown in Figure 7, and aircraft in the two situations can produce the same noise and RCS.

Therefore, a combined parameter called scissors angle difference is set up for coaxial tilt-rotor aircraft, reflecting the phase difference of the two coaxial rotors. The scissors angle difference is defined as:

(34) $$\Delta \chi =\chi_{right}-\chi_{left}$$

### 3.3. Simulation

**Figure 8:**
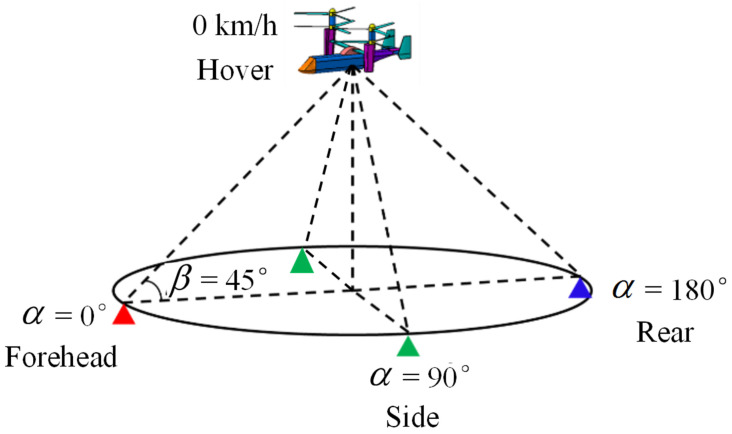
Schematic diagram of simulation in helicopter mode.

**Figure 9:**
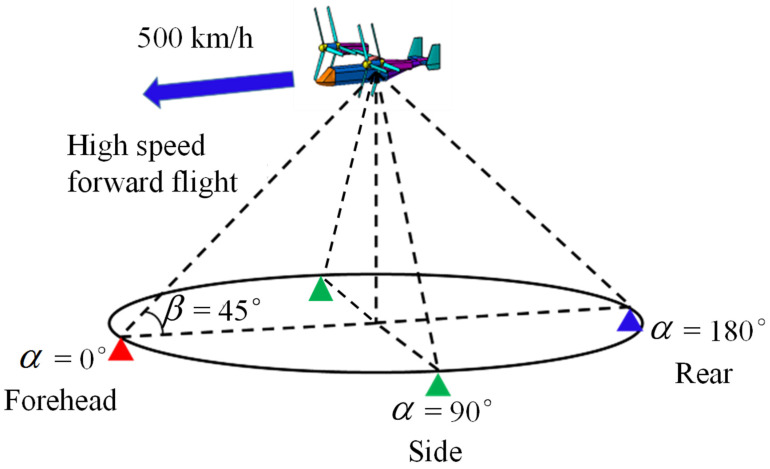
Schematic diagram of simulation in fixed-wing mode.

In this paper, simulations of coaxial tilt-rotor aircraft in helicopter mode and fixed-wing mode are carried out for the exploration of the noise and dynamic RCS characteristics. In the simulation cases shown in Figure 8 and Figure 9, the detector receives both acoustic noise and scattered radar waves from coaxial tilt-rotor aircraft at the elevation angle β = 45° and the azimuth angle at α = 0° (forehead), 90° (side) and 180° (rear). The distance from the helicopter to the detector is 1000 m. In helicopter mode, the speed of the aircraft is set to 0, illustrating the status of hovering. In fixed-wing mode, the speed of the aircraft is set to 139 m/s (500 km/h), illustrating the status of high speed forward flight.

## 4. Results and Discussion

As for the results of previous research, only a few conditions have been taken into consideration, and the influences of rotor phase parameters on RCS and noise SPL cannot be observed entirely. Therefore, carpet diagrams showing the distribution of RCS and noise SPL under different CSM parameter combinations are introduced in this paper, and the optimized CSM parameter combinations (design points) can be selected from the carpet diagrams.

In this paper, 12 cases are considered for the calculation of the noise SPL and dynamic RCS of coaxial tilt-rotor aircraft at different azimuth angles and flight modes. Six of the cases are carried out for noise SPL calculation, while the other six of the cases are for dynamic RCS calculation. The various parameters of the 12 cases are summarized as follows, which can be seen in Table 3. Moreover, some fixed parameters that were constant in all of the cases are listed in Table 4.

### 4.1. Carpet Diagrams of Noise SPL Values in Helicopter Mode

#### 4.1.1. Noise SPL Values at an Azimuth Angle = 0° (Forehead)

**Figure 10:**
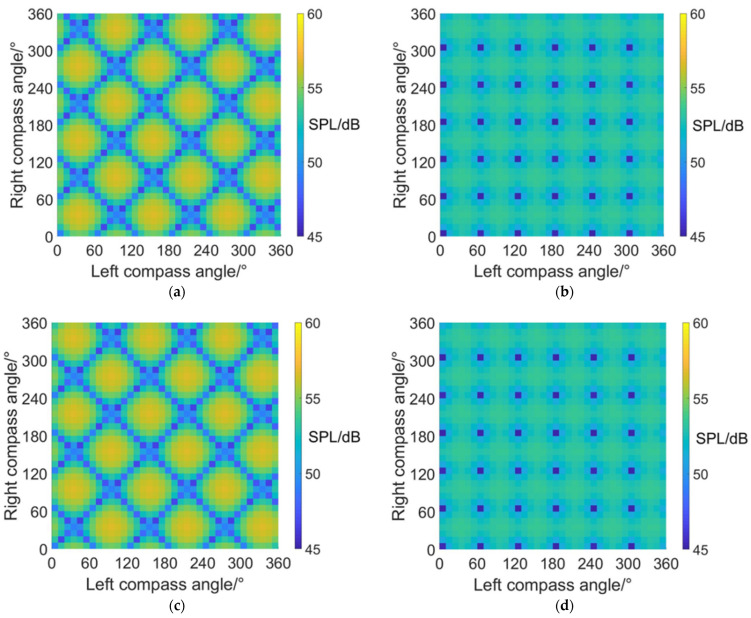
Carpet diagrams of noise SPL in helicopter mode at an azimuth angle = 0° with the scissors angle difference = (**a**) 0°, (**b**) 30°, (**c**) 60°, (**d**) 90°.

Figure 10 shows the noise SPL distributions on carpet diagrams with scissors angle difference = 0°, 30°, 60° and 90° at an azimuth angle = 0° (forehead) in helicopter mode. It can be observed from Figure 10 that the variation of the scissors angle difference has influences on both the distribution and values of noise SPL. When the scissors angle difference equals 0° and 60°, the relatively low values (blue color bars) form obviously visible lines in carpet diagrams. However, the relatively low values only emerge as separated points when the scissors angle difference equals 30° and 90°.

With scissors angle difference = 30° or 90°, noise SPL reaches relatively low values when left and right compass angles satisfy the relationship of:

(35) $$\left\{ \begin{array}{l} \psi_{left}=k_{1}\cdot \frac{\pi }{3}\\ \psi_{right}=k_{2}\cdot \frac{\pi }{3} \end{array}\right.\left(k_{1},k_{2}\in Z\right)$$

With scissors angle difference = 0°, noise SPL reaches relatively low values when left and right compass angles satisfy the relationship of:

(36) $$\psi_{right}=k\cdot \frac{\pi }{3}+{\left(-1\right)}^{k+1}\cdot \psi_{left}\left(k\in Z\right)$$

With scissors angle difference = 60°, noise SPL reaches relatively low values when left and right compass angles satisfy the relationship of:

(37) $$\psi_{right}=k\cdot \frac{\pi }{3}+{\left(-1\right)}^{k}\cdot \psi_{left}\left(k\in Z\right)$$

The highest SPL and range of relatively low SPL values at an azimuth angle = 0° in helicopter mode are listed in Table 5, indicating that the noise SPL at an azimuth angle = 0° (forehead) can be reduced by 7.4~10.4 dB after CSM phase modulation.

#### 4.1.2. Noise SPL Values at an azimuth Angle = 90° (Side)

**Figure 11:**
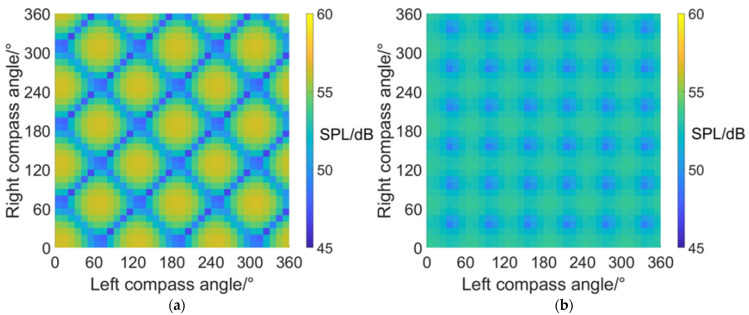

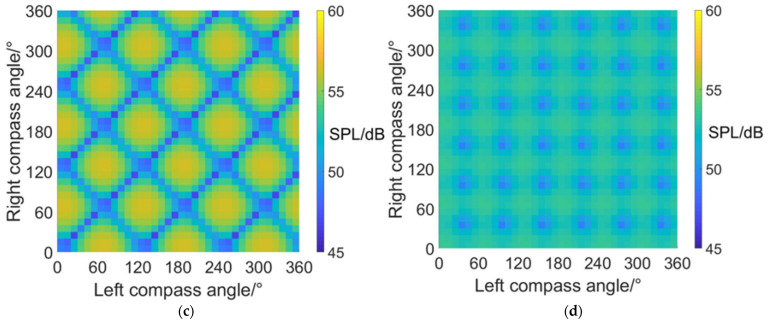
Carpet diagrams of noise SPL in helicopter mode at an azimuth angle = 90° with a scissors angle difference = (**a**) 0°, (**b**) 30°, (**c**) 60°, (**d**) 90°.

Figure 11 shows the noise SPL distributions on carpet diagrams with scissors angle difference = 0°, 30°, 60° and 90° at an azimuth angle = 90° (side) in helicopter mode. It can be observed from Figure 11 that the distribution images of noise SPL values on carpet diagrams have similar shapes but different positions, with the same scissors angle difference. The relatively low values (blue color bars) also form obviously visible lines when scissors angle difference = 0° or 60° and are separated into points when scissors angle difference = 30° or 90°. However, the positions are different compared with Figure 10.

When the scissors angle difference = 30° or 90°, noise SPL reaches relatively low values when left and right compass angles satisfy the relationship of:

(38) $$\left\{ \begin{array}{l} \psi_{left}=\frac{\pi }{6}+k_{1}\cdot \frac{\pi }{3}\\ \psi_{right}=\frac{\pi }{6}+k_{2}\cdot \frac{\pi }{3} \end{array}\right.\left(k_{1},k_{2}\in Z\right)$$

When the scissors angle difference = 0°, noise SPL reaches relatively low values when left and right compass angles satisfy the relationship of:

(39) $$\psi_{right}=\left(2k+1\right)\cdot \frac{\pi }{3}\pm \psi_{left}\left(k\in Z\right)$$

When the scissors angle difference = 60°, noise SPL reaches relatively low values when left and right compass angles satisfy the relationship of:

(40) $$\psi_{right}=2k\cdot \frac{\pi }{3}\pm \psi_{left}\left(k\in Z\right)$$

The maximum and minimum SPL values at an azimuth angle = 90° in helicopter mode are listed in Table 6, indicating that the noise SPL at an azimuth angle = 90° (side) can be reduced by 9.6 dB at most after CSM phase modulation.

#### 4.1.3. Noise SPL Values at an Azimuth Angle = 180° (Rear)

**Figure 12:**
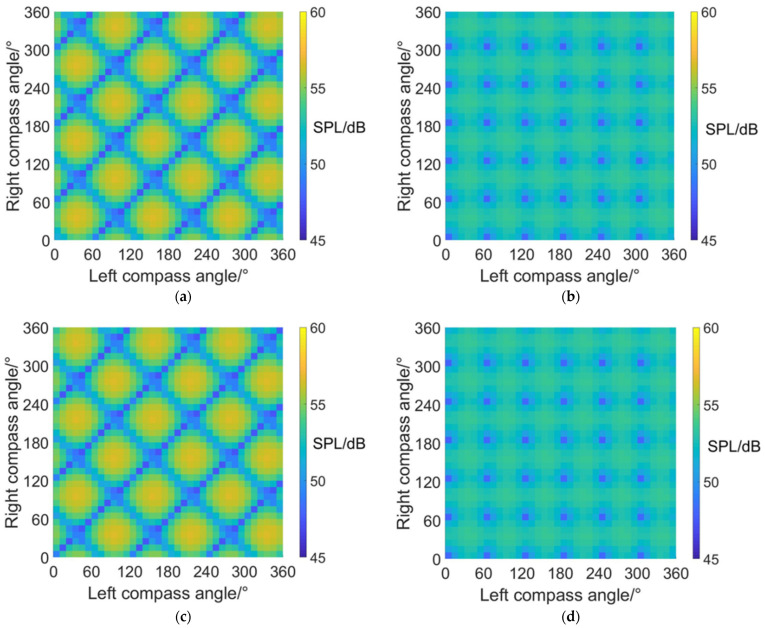
Carpet diagrams of noise SPL in helicopter mode at an azimuth angle = 180° with a scissors angle difference = (**a**) 0°, (**b**) 30°, (**c**) 60°, (**d**) 90°.

Figure 12 shows the noise SPL distributions on carpet diagrams with a scissors angle difference = 0°, 30°, 60° and 90° at an azimuth angle = 180° (rear) in helicopter mode. Compared with Figure 10, it can be discovered that the distributions of noise SPL values have almost the same shape and position at an azimuth = 0° and 180, and left and right compass angles also satisfy the relationship of Equations (39) and (40) so that noise SPL can reach relatively low values. Table 7 shows the maximum and minimum SPL values at an azimuth angle = 90° in helicopter mode. Compared with Table 6, the noise SPL values at azimuth angles = 0° and 180° are also close to each other.

### 4.2. Carpet Diagrams of Noise SPL Values in Fixed-Wing Mode

**Figure 13:**
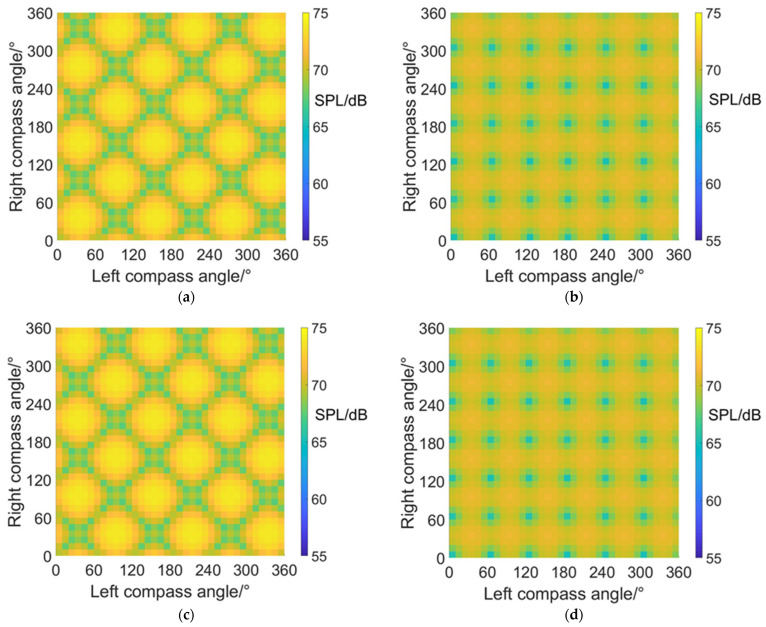
Carpet diagrams of noise SPL in fixed-wing mode at an azimuth angle = 0° with scissors angle difference = (**a**) 0°, (**b**) 30°, (**c**) 60°, (**d**) 90°.

**Figure 14:**
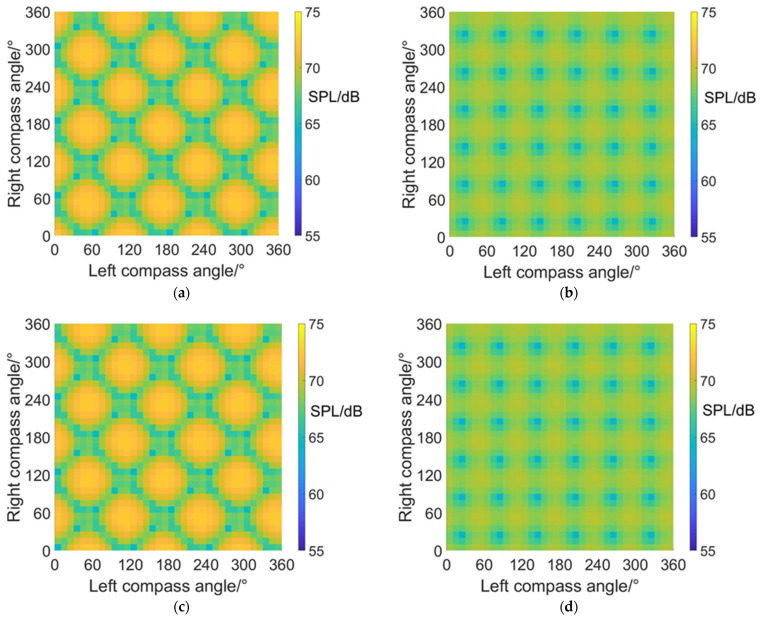
Carpet diagrams of noise SPL in fixed-wing mode at an azimuth angle = 90° with scissors angle difference = (**a**) 0°, (**b**) 30°, (**c**) 60°, (**d**) 90°.

**Figure 15:**
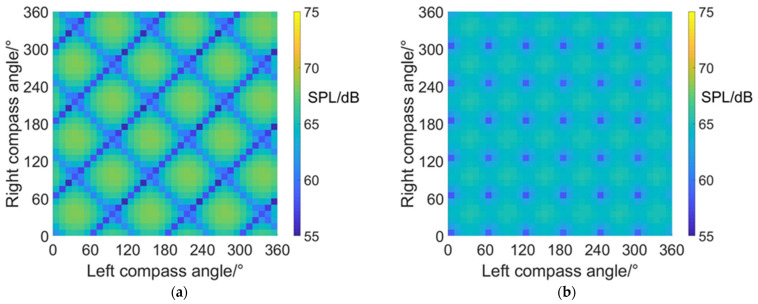

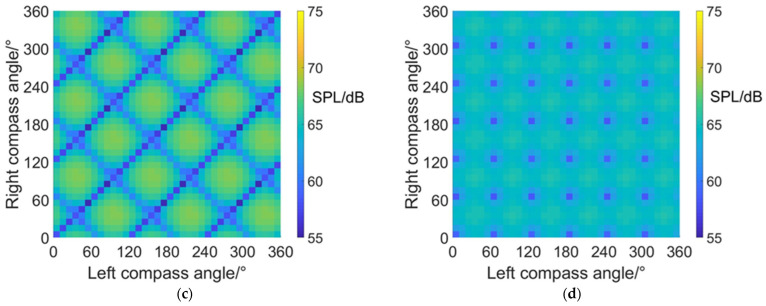
Carpet diagrams of noise SPL in fixed-wing mode at an azimuth angle = 180° with scissors angle difference = (**a**) 0°, (**b**) 30°, (**c**) 60°, (**d**) 90°.

The noise SPL distributions on carpet diagrams with scissors angle difference = 0°, 30°, 60° and 90° at azimuth angles = 0° (forehead), 90° (side) and 180° (rear) in fixed-wing mode are shown in Figure 13, Figure 14 and Figure 15. According to the comparisons of noise SPL carpet diagrams in helicopter mode and fixed-wing mode, it can be indicated that the distribution rules of SPL values on carpet diagrams are similar at the same scissors angle difference, but the SPL values in helicopter mode and fixed-wing mode are quite different. The noise SPL values for all azimuth angles in fixed-wing mode are listed in Table 8, Table 9 and Table 10, indicating that the noise produced by coaxial tilt-rotor aircraft in fixed-wing mode is more significant than that in helicopter mode. Moreover, it can be discovered from Figure 13, Figure 14 and Figure 15 that the noise SPL values at an azimuth angle = 0° are obviously higher than those at an azimuth angle = 90° and 180°. The reason for this is that when the coaxial-tilt aircraft maintains high-speed forward flight, the air compression at the forehead is more significant than that at the side and rear, therefore generating higher noise.

### 4.3. Carpet Diagrams of Average RCS in Helicopter Mode

The calculated dynamic RCS of a coaxial tilt-rotor aircraft consists of the RCS values at all of the time points in a period of time, and the RCS values change as time flows. Average RCS in one period of rotor rotation is calculated in order to give a measurement of the RCS level of coaxial tilt-rotor aircraft at a specific azimuth angle and flight status, and carpet diagrams of average RCS are put forward in this paper.

#### 4.3.1. Average RCS Values at an Azimuth Angle = 0° (Forehead)

**Figure 16:**
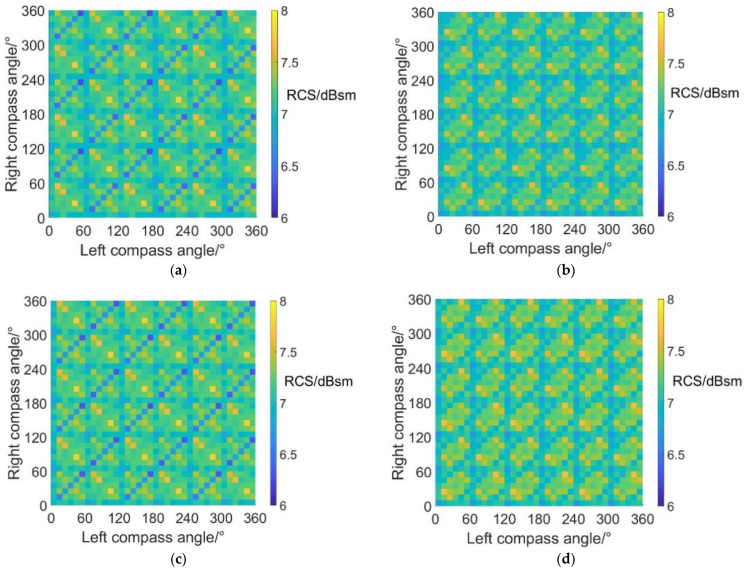
Carpet diagrams of average RCS in helicopter mode at an azimuth angle = 0° with scissors angle difference = (**a**) 0°, (**b**) 30°, (**c**) 60°, (**d**) 90°.

The carpet diagrams of average RCS in helicopter mode at an azimuth angle = 0° can be seen in Figure 16. It can be observed that the scissors angle difference can influence the distribution as well as the size of the RCS values on the carpet diagrams. Similar to the distributions of SPL values, the relatively low RCS values (dark blue color bars) also form obviously visible lines when scissors angle difference = 0° and 60° and also are relatively separated into points when scissors angle difference = 30° and 90°. Moreover, the average RCS values in helicopter mode at an azimuth angle = 0° also reach relatively low values when left and right compass angles satisfy the relationship of Equation (38) when the scissors angle = 30° or 90°, Equation (39) when the scissors angle = 0° and Equation (40) when the scissors angle = 60°.

The maximum and minimum average RCS values at an azimuth angle = 0° in helicopter mode is listed in Table 11, indicating that the noise SPL at an azimuth angle = 0° (forehead) can be reduced by 1.5 dBsm at most after CSM phase modulation.

#### 4.3.2. Average RCS Values at an Azimuth Angle = 90° (Side)

**Figure 17:**
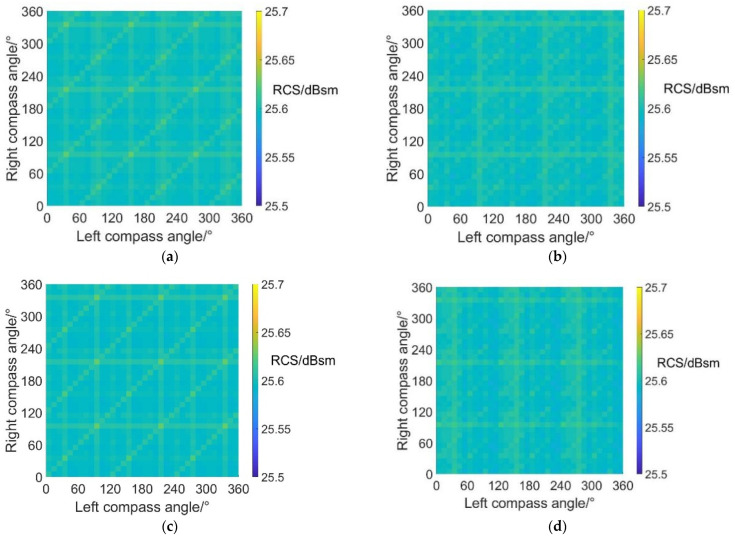
Carpet diagrams of average RCS in helicopter mode at an azimuth angle = 90° with scissors angle difference = (**a**) 0°, (**b**) 30°, (**c**) 60°, (**d**) 90°.

It can be noticed from Figure 17 and Table 12 that the average RCS values in helicopter mode at an azimuth angle = 90° are always close to 25.6 dBsm (green and light blue color bars). The maximum average RCS value reaches 25.6259 dBsm, while the minimum average RCS value reaches 25.5849 dBsm, with a variation range of only 0.041 dBsm. In addition, the average RCS values observed from the sides are much higher than those observed from the head and from the rear. However, the average RCS observed from the side remains almost the same value despite the variations in compass angles and scissors angle differences. The reasons can be explained by the radar scattering effect of the fuselage, which appears to be more significant in the side directions, leading to higher RCS values. Moreover, the existence of the fuselage brings shadow effects to the coaxial rotors, reducing the influence of compass angles and scissors angle difference on dynamic RCS. Compared with Figure 11, it can be inferred that the optimization of compass angles and scissors angle differences mainly contributes to the reduction of noise.

#### 4.3.3. Average RCS Values at an Azimuth Angle = 180° (Rear)

**Figure 18:**
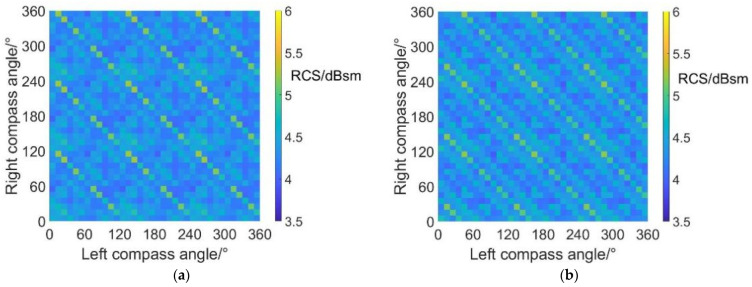

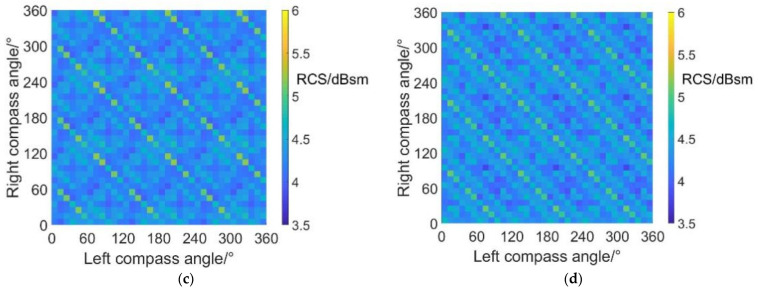
Carpet diagrams of average RCS in helicopter mode at an azimuth angle = 180° with scissors angle difference = (**a**) 0°, (**b**) 30°, (**c**) 60°, (**d**) 90°.

Figure 18 shows the carpet diagrams of average RCS in helicopter mode at an azimuth angle = 180°. It can be seen that the relatively high RCS values (yellow and green color bars) form clear lines while the relatively low RCS values (blue color bars) are rather separated. However, the vague lines formed by the relatively low RCS values can still be roughly seen in Figure 18a,c, with scissors angle difference = 0° and 60°. The vague lines indicate that average RCS can also reach relatively low values when left and right compass angles satisfy the relationship of Equation (39) at scissors angle difference = 0°, and Equation (40) at scissors angle difference = 60°. The average RCS values are about 4.1~4.2 dBsm, which are also relatively low values in the carpet diagrams. As for the situation when the scissors angle difference = 30° and 90°, the noise SPL can reach relatively low values when left and right compass angles satisfy the relationship of Equation (38). However, the average RCS reaches approximately 4.7 dBsm, represented by the light green bars (not relatively low values) in the carpet diagrams. Therefore, the noise SPL and the average RCS at an azimuth angle = 180° (rear) can reach relatively low values at the same time when the scissors angle difference = 0° and 60° but cannot when the scissors angle difference = 0° and 60°.

The maximum and minimum average RCS values in helicopter mode at an azimuth angle = 180° are listed in Table 13. Compared with Table 11 and Table 12, the minimum average RCS in helicopter mode at an azimuth angle = 180° appears to be lower than that at an azimuth angle = 0° and 90°. The reason can be explained by the fact that the radar scattering effect of the tail is weaker than that of the cabin at the forehead and much weaker than the side face of the fuselage.

### 4.4. Carpet Diagrams of Average RCS in Fixed-Wing Mode

#### 4.4.1. Average RCS Values at an Azimuth Angle = 0° (Forehead)

**Figure 19:**
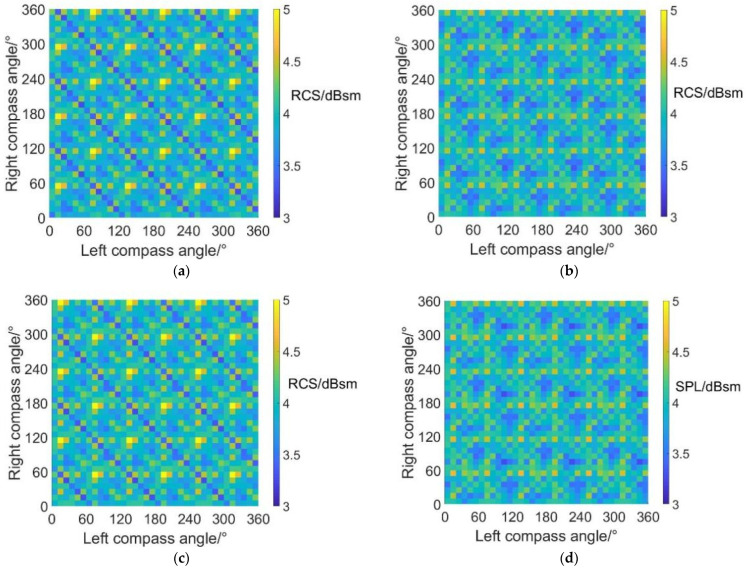
Carpet diagrams of average RCS in fixed-wing mode at an azimuth angle = 0° with scissors angle difference = (**a**) 0°, (**b**) 30°, (**c**) 60°, (**d**) 90°.

The carpet diagrams of average RCS in fixed-wing mode at an azimuth angle = 0° can be seen in Figure 19. Analogously, the relatively low RCS values (dark blue color bars) also form obviously visible lines when the scissors angle difference = 0° and 60°, and also relatively separate points when the scissors angle difference = 30° and 90°. However, the directions of the lines in Figure 19a,c is vertical to the directions of the lines in Figure 16a,c. Therefore, in fixed-wing mode at an azimuth angle = 0°, the average RCS can achieve relatively low values when left and right compass angles satisfy the relationship of:

(41) $$\psi_{right}=2k\cdot \frac{\pi }{3}-\psi_{left}\left(k\in Z\right)$$

at scissors angle difference = 0°.

When the scissors angle difference = 60°, left and right compass angles should satisfy the relationship of:

(42) $$\psi_{right}=\left(2k+1\right)\cdot \frac{\pi }{3}-\psi_{left}\left(k\in Z\right)$$

The points illustrated in Equation (41) are included in those illustrated in Equation (39), and the points illustrated in Equation (42) are included in those illustrated in Equation (40), indicating that the noise SPL and average RCS can reach relatively low values at the same time when the scissors angle difference = 0° and 60°.

When the scissors angle difference = 30° and 90°, noise SPL can reach relatively low values when the left and right compass angles satisfy the relationship of Equation (38). However, the average RCS values have a range of 4.0~4.2 dBsm, green bars in Figure 19b,d, not the relatively low RCS values.

The maximum and minimum average RCS values in fixed-wing mode at an azimuth angle = 0° are listed in Table 14. By CSM phase modulation, the average RCS in fixed-wing mode at an azimuth angle = 0° can be reduced by 1.8 dBsm at most. Combined with Figure 13, it can be indicated that the situations of scissors angle difference = 0° and 60° might be better for RCS reduction as well as noise reduction.

#### 4.4.2. Average RCS Values at an Azimuth Angle = 90° (Side)

**Figure 20:**
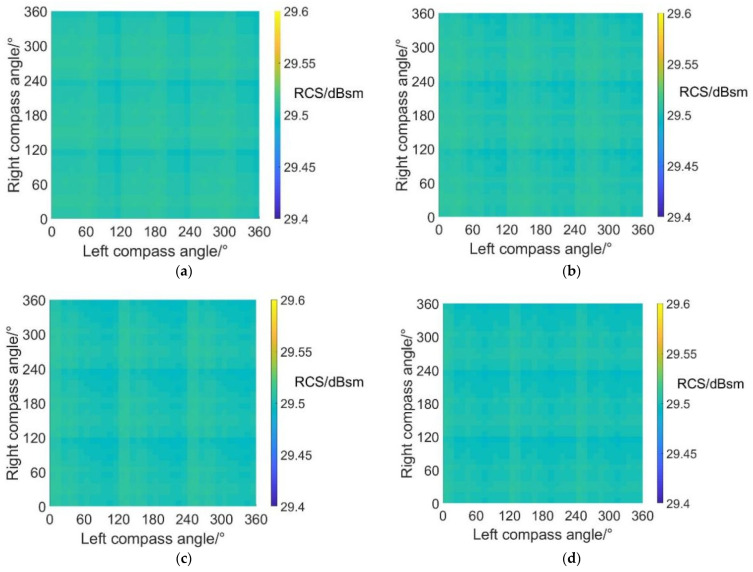
Carpet diagrams of average RCS in fixed-wing mode at an azimuth angle = 90° with scissors angle difference = (**a**) 0°, (**b**) 30°, (**c**) 60°, (**d**) 90°.

Figure 20 shows the carpet diagrams of average RCS in fixed-wing mode at an azimuth angle = 90° and the maximum and minimum values are listed in Table 15. Similar to the situation in helicopter mode, the average RCS values in fixed-wing mode at an azimuth angle = 90° are always close to 29.5 dBsm, remaining almost the same. The maximum average RCS value reaches 29.5128 dBsm, while the minimum average RCS value reaches 29.4957 dBsm, with a variation range of only 0.0171 dBsm. Moreover, the average RCS values in fixed-wing mode at an azimuth angle = 90° are generally higher than those in helicopter mode. The reason can be explained by the fact that the scattering effects of cabins are more significant under the fixed-wing mode because the projection areas of cabins are larger.

#### 4.4.3. Average RCS Values at an Azimuth Angle = 180° (Rear)

**Figure 21:**
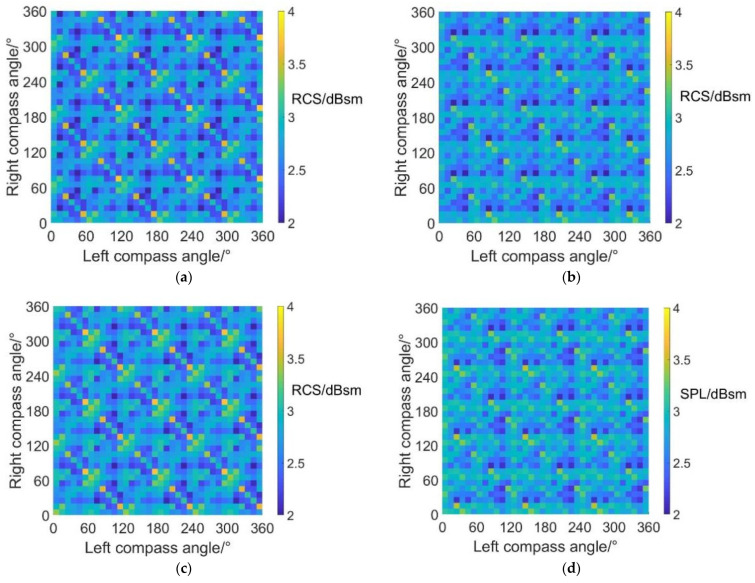
Carpet diagrams of average RCS in fixed-wing mode at an azimuth angle = 180° with scissors angle difference = (**a**) 0°, (**b**) 30°, (**c**) 60°, (**d**) 90°.

Figure 21 shows the carpet diagrams of average RCS in fixed-wing mode at an azimuth angle = 180°. Similarly, the relatively high values form obviously visible lines, while the relatively low values are separated into points. Fortunately for the situations of scissors angle difference = 0° and 60°, when left and right compass angles satisfy the relationship of Equations (39) and (40) in order to obtain relatively low noise SPL, the lines formed by relatively high average RCS values can also be avoided, making contributions to the reduction of RCS at an azimuth angle = 180°. As for the situations where the scissors angle difference = 30° and 90°, the average RCS reaches 3 dBsm when compass angles satisfy Equation (38).

The maximum and minimum average RCS values in fixed-wing mode at an azimuth angle = 180° are listed in Table 16. By CSM phase modulation, the average RCS in fixed-wing mode at an azimuth angle = 180° can be reduced by 1.8 dBsm at most.

### 4.5. Optimal CSM Phase Parameters

In order to achieve the goal that both noise SPL and average RCS reach relatively low values, proper CSM phase parameters, including compass angles (left and right) and scissors angle difference, should be selected. Considering that coaxial tilt-rotor aircraft may switch from helicopter mode to fixed-wing mode or from fixed-wing mode to helicopter mode, optimal CSM phase parameters should be determined for the reduction of noise and RCS in both of the two modes.

#### 4.5.1. Optimal CSM Phase Parameters at an Azimuth Angle = 0° (Forehead)

At scissors angle difference = 0°, and combining Equations (39) and (41), the relationship of left and right compass angles should be:

(43) $$\left\{ \begin{array}{l} \psi_{left}=\frac{\pi }{6}+2k_{1}\cdot \frac{\pi }{3}\\ \psi_{right}=\frac{\pi }{2}+2k_{2}\cdot \frac{\pi }{3} \end{array}\right.\left(k_{1},k_{2}\in Z\right)$$

At scissors angle difference = 30° and considering that the variations in noise SPL are greater than average RCS, the relationship of left and right compass angles should be:

(44) $$\left\{ \begin{array}{l} \psi_{left}=k_{1}\cdot \frac{\pi }{3}\\ \psi_{right}=k_{2}\cdot \frac{\pi }{3} \end{array}\right.\left(k_{1},k_{2}\in Z\right)$$

At scissors angle difference = 60° and combining Equations (40) and (42), the relationship of left and right compass angles should be:

(45) $$\left\{ \begin{array}{l} \psi_{left}=\frac{\pi }{6}+2k_{1}\cdot \frac{\pi }{3}\\ \psi_{right}=\frac{\pi }{6}+2k_{2}\cdot \frac{\pi }{3} \end{array}\right.\left(k_{1},k_{2}\in Z\right)$$

At scissors angle difference = 90°, the relationship between left and right compass angles should be:

(46) $$\left\{ \begin{array}{l} \psi_{left}=k_{1}\cdot \frac{\pi }{3}\\ \psi_{right}=k_{2}\cdot \frac{\pi }{3} \end{array}\right.\left(k_{1},k_{2}\in Z\right)$$

With the CSM phase parameters of Equations (43)–(46), the noise SPL and average RCS values are listed in Table 17.

#### 4.5.2. Optimal CSM Phase Parameters at an Azimuth Angle = 90° (Side)

The dynamic RCS remains almost the same value at an azimuth angle = 90° in either helicopter mode or fixed-wing mode. Therefore, we should pay attention to the reduction of noise SPL.

At scissors angle difference = 0°, the relationship between left and right compass angles should be:

(47) $$\psi_{right}=\left(2k+1\right)\cdot \frac{\pi }{3}\pm \psi_{left}\left(k\in Z\right)$$

At scissors angle difference = 30°, the relationship between left and right compass angles should be:

(48) $$\left\{ \begin{array}{l} \psi_{left}=\frac{\pi }{6}+k_{1}\cdot \frac{\pi }{3}\\ \psi_{right}=\frac{\pi }{6}+k_{2}\cdot \frac{\pi }{3} \end{array}\right.\left(k_{1},k_{2}\in Z\right)$$

At scissors angle difference = 60°, the relationship between left and right compass angles should be:

(49) $$\psi_{right}=\left(2k+1\right)\cdot \frac{\pi }{3}\pm \psi_{left}\left(k\in Z\right)$$

At scissors angle difference = 90°, the relationship between left and right compass angles should be:

(50) $$\left\{ \begin{array}{l} \psi_{left}=\frac{\pi }{6}+k_{1}\cdot \frac{\pi }{3}\\ \psi_{right}=\frac{\pi }{6}+k_{2}\cdot \frac{\pi }{3} \end{array}\right.\left(k_{1},k_{2}\in Z\right)$$

With the CSM phase parameters of Equations (47)–(50), the noise SPL and average RCS values are listed in Table 18.

#### 4.5.3. Optimal CSM Phase Parameters at an Azimuth Angle = 180° (Rear)

The distribution rules of dynamic RCS at an azimuth angle = 180° are rather vague, according to Figure 18 and Figure 21. Considering that the dynamic RCS values and fixed-wing noise SPL values at an azimuth angle = 180° are obviously lower than those at an azimuth angle = 0° and 90°, the reduction of noise SPL at an azimuth angle = 180° in helicopter mode should be given the most attention. Therefore:

At scissors angle difference = 0°, noise SPL reaches relatively low values when left and right compass angles satisfy the relationship of:

(51) $$\psi_{right}=k\cdot \frac{\pi }{3}+{\left(-1\right)}^{k+1}\cdot \psi_{left}\left(k\in Z\right)$$

At scissors angle difference = 30°, noise SPL reaches relatively low values when left and right compass angles satisfy the relationship of:

(52) $$\left\{ \begin{array}{l} \psi_{left}=k_{1}\cdot \frac{\pi }{3}\\ \psi_{right}=k_{2}\cdot \frac{\pi }{3} \end{array}\right.\left(k_{1},k_{2}\in Z\right)$$

At scissors angle difference = 60°, noise SPL reaches relatively low values when left and right compass angles satisfy the relationship of:

(53) $$\psi_{right}=k\cdot \frac{\pi }{3}+{\left(-1\right)}^{k}\cdot \psi_{left}\left(k\in Z\right)$$

At scissors angle difference = 90°, noise SPL reaches relatively low values when left and right compass angles satisfy the relationship of:

(54) $$\left\{ \begin{array}{l} \psi_{left}=k_{1}\cdot \frac{\pi }{3}\\ \psi_{right}=k_{2}\cdot \frac{\pi }{3} \end{array}\right.\left(k_{1},k_{2}\in Z\right)$$

With the CSM phase parameters of Equations (51)–(54), the noise SPL and average RCS values are listed in Table 19.

#### 4.5.4. Optimal CSM Parameters

According to the results above, it can be discerned that:

(a) The points represented by Equations (43) and (47) are contained by those represented by Equation (51), and the points represented by Equations (45) and (49) are also contained by those represented by Equation (53). This indicates that with scissors angle difference = 0° or 60°, noise SPL and average RCS can reach relatively low values at the same time.

At scissors angle difference = 0°, the optimal CSM phase parameters should be:

(55) $$\left\{ \begin{array}{l} \psi_{left}=\frac{\pi }{6}+2k_{1}\cdot \frac{\pi }{3}\\ \psi_{right}=\frac{\pi }{2}+2k_{2}\cdot \frac{\pi }{3}\\ \Delta \chi =0 \end{array}\right.\left(k_{1},k_{2}\in Z\right)$$

At scissors angle difference = 60°, the optimal CSM phase parameters should be:

(56) $$\left\{ \begin{array}{l} \psi_{left}=\frac{\pi }{6}+2k_{1}\cdot \frac{\pi }{3}\\ \psi_{right}=\frac{\pi }{6}+2k_{2}\cdot \frac{\pi }{3}\\ \Delta \chi =\frac{\pi }{3} \end{array}\right.\left(k_{1},k_{2}\in Z\right)$$

**Figure 22:**
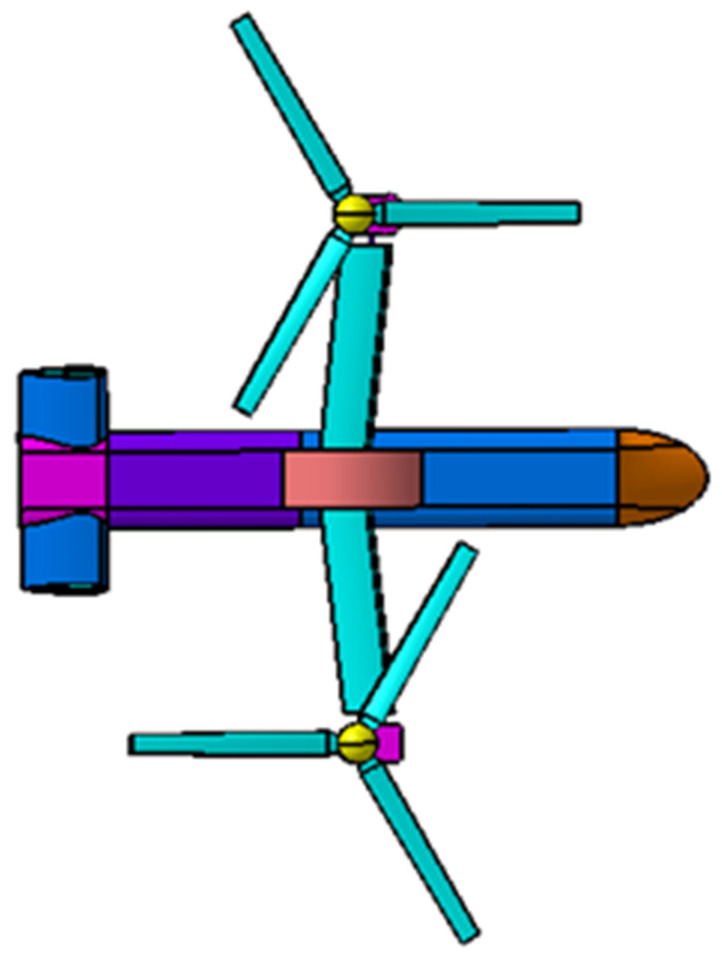
View of a coaxial tilt-rotor aircraft with PPC1.

According to the periodicity and symmetry of coaxial rotors, the two CSM phase parameter combinations represent the same condition for coaxial tilt-rotor aircraft, as is shown in Figure 22. The noise SPL and average RCS values are listed in Table 20. The CSM phase parameter combinations of Equations (55) and (56) are named as Phase Parameter Combination 1 (PPC1).

(b) Equations (44), (46), (52) and (54) are the same, but conflict with Equations (48) and (50). This illustrates that we have to make a choice between the forehead/rear and side when the scissors angle difference = 30° or 90°.

With priority given to noise and RCS reduction of the forehead/rear at a scissors angle difference = 30°, the optimal CSM phase parameters should be:

(57) $$\left\{ \begin{array}{l} \psi_{left}=k_{1}\cdot \frac{\pi }{3}\\ \psi_{right}=k_{2}\cdot \frac{\pi }{3}\\ \Delta \chi =\frac{\pi }{6} \end{array}\right.\left(k_{1},k_{2}\in Z\right)$$

With priority given to noise and RCS reduction of the side at a scissors angle difference = 30°, the optimal CSM phase parameters should be:

(58) $$\left\{ \begin{array}{l} \psi_{left}=\frac{\pi }{6}+k_{1}\cdot \frac{\pi }{3}\\ \psi_{right}=\frac{\pi }{6}+k_{2}\cdot \frac{\pi }{3}\\ \Delta \chi =\frac{\pi }{6} \end{array}\right.\left(k_{1},k_{2}\in Z\right)$$

With priority given to noise and RCS reduction of the forehead/rear at a scissors angle difference = 90°, the optimal CSM phase parameters should be:

(59) $$\left\{ \begin{array}{l} \psi_{left}=k_{1}\cdot \frac{\pi }{3}\\ \psi_{right}=k_{2}\cdot \frac{\pi }{3}\\ \Delta \chi =\frac{\pi }{2} \end{array}\right.\left(k_{1},k_{2}\in Z\right)$$

With priority given to noise and RCS reduction of the side at a scissors angle difference = 90°, the optimal CSM phase parameters should be:

(60) $$\left\{ \begin{array}{l} \psi_{left}=\frac{\pi }{6}+k_{1}\cdot \frac{\pi }{3}\\ \psi_{right}=\frac{\pi }{6}+k_{2}\cdot \frac{\pi }{3}\\ \Delta \chi =\frac{\pi }{2} \end{array}\right.\left(k_{1},k_{2}\in Z\right)$$

**Figure 23:**
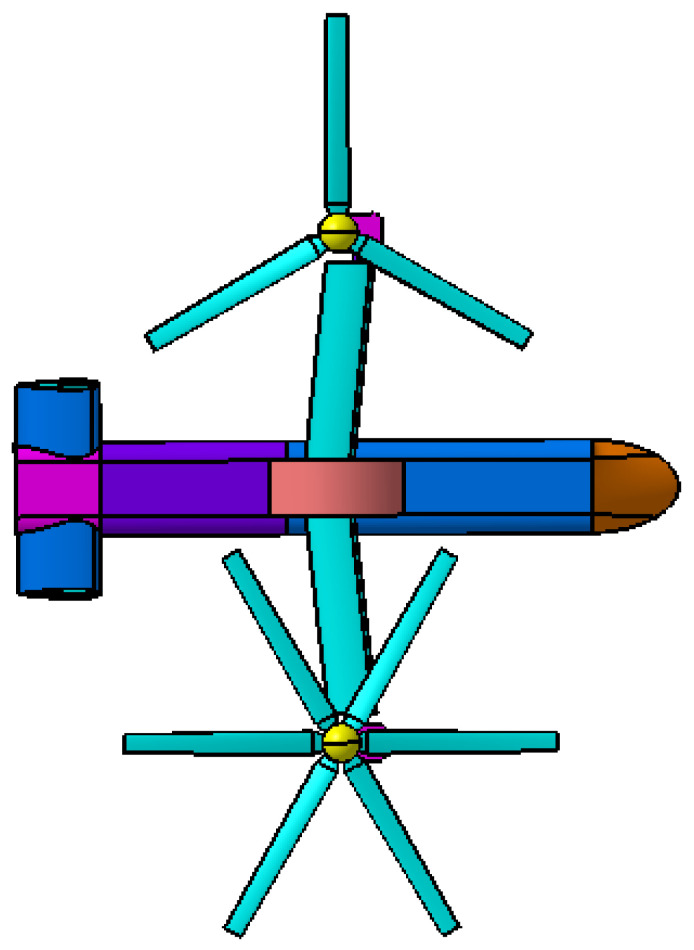
View of a coaxial tilt-rotor aircraft with PPC2.

**Figure 24:**
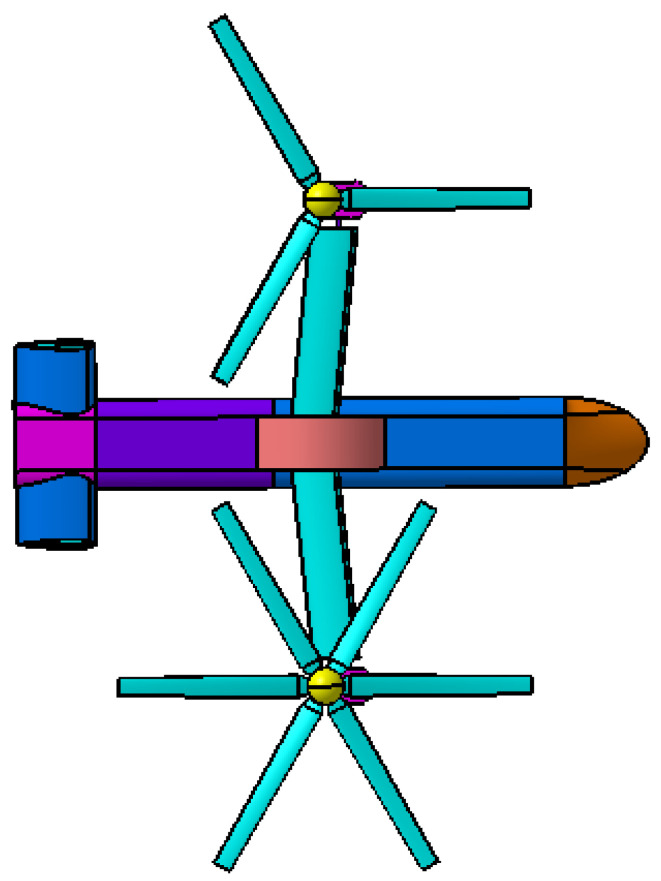
View of a coaxial tilt-rotor aircraft with PPC3.

Ignoring the influence of the distance between the lower and upper rotors of a coaxial rotor, the CSM phase parameter combinations Equations (57) and (59) represent the same status of aircraft, as is shown in Figure 23. The noise SPL and average RCS values are listed in Table 21. Similarly, CSM phase parameter combinations Equations (58) and (60) represent another status of aircraft shown in Figure 24, and Table 22 shows the noise SPL and average RCS values. The CSM phase parameter combinations of Equations (57) and (59) are named as Phase Parameter Combination 2 (PPC2), while the CSM phase parameter combinations of Equations (58) and (60) are named as Phase Parameter Combination 3 (PPC3).

According to the comparisons of Table 20, Table 21 and Table 22, the average RCS values of the three PPCs have little difference with each other, but the noise SPL values obviously vary from each other. The design with PPC2 has a lower noise SPL at an azimuth angle = 0° (forehead) and 180° (Rear), while the design with PPC3 has a lower noise SPL at an azimuth angle = 90° (side). Table 23 shows the reduction values (compared with maximum values) of noise SPL and average RCS of PPC1, PPC2 and PPC3.

It can be seen in Table 23 that different phase parameter combinations (PPCs) can reach different noise and RCS reduction values at specific azimuth angles or flight modes:

(1) At an azimuth angle = 0°, the maximum noise reduction reaches 11.4 dB in helicopter mode and 8.9 dB in fixed-wing mode, and both of them correspond to PPC2. The maximum RCS reduction reaches 1.1 dBsm in helicopter mode and 1.6 dBsm in fixed-wing mode, and both of them correspond to PPC1. By the comparisons between PPC1 and PPC2, it can be obtained that at an azimuth angle = 0° PPC1 makes most contribution to RCS reduction in fixed-wing mode, and PPC2 makes the biggest contribution to noise reduction in both flight modes.
(2) At an azimuth angle = 90°, the maximum noise reduction reaches 15.1 dB in helicopter mode and 8.9 dB in fixed-wing mode, and both of them correspond to PPC3. The RCS reduction is always 0 in all of the phase parameter combinations (PPCs). It can be determined that at an azimuth angle = 90°, PPC3 makes the biggest contribution to noise reduction in both flight modes but almost no contribution to RCS reduction.
(3) At an azimuth angle = 180°, the maximum noise reduction reaches 11.4 dB in helicopter mode and 10.1 dB in fixed-wing mode; 11.4 dB corresponds to PPC2, and 10.1 dB corresponds to PPC1. The maximum RCS reduction reaches 1.2 dBsm in helicopter mode and 1 dBsm in fixed-wing mode, and both of them correspond to PPC1. It can be determined that at an azimuth angle =180°, PPC1 makes the biggest contribution to noise reduction in fixed-wing mode and to RCS reduction in both flight modes. PPC2 makes the biggest contribution to noise reduction in helicopter mode.

According to the analysis above, it can be concluded that the stealth design with PPC1 can contribute most to RCS reduction, the stealth design with PPC2 can contribute most to noise reduction at the forehead and rear, and the stealth design with PPC3 can contribute most to noise reduction at the side.

## 5. Conclusions

In this paper, an approach to the prediction of both the sound pressure level (SPL) of noise and the dynamic RCS of coaxial-tilt aircraft is carried out based on the theories of FW-H equation. A dynamic RCS and noise prediction and reduction method for coaxial tilt-rotor aircraft based on phase modulation is established with the generated rotation matrix (GRM), compass-scissors model (CSM) and carpet diagrams put forward.

Compared with the existing work, the establishment of GRM gives a universal formula for the time-domain grid coordinate transformation of all kinds of rotation parts with arbitrary rotation centers and rotation axis directions. As a newly established model, CSM expresses the description of the orientation and the rotation phase status of coaxial rotors, making research on the phase characteristics of coaxial rotors possible. Carpet diagrams applied in this paper intuitively show the variations of noise SPL and dynamic RCS under different phase parameters (defined in CSM) and can be helpful to the stealth design of tilt-coaxial rotor aircraft using phase modulation of the coaxial rotors. According to the results, the following conclusions can be obtained:

(1) The generated rotation matrix (GRM) has the ability to give a concise expression of the time-domain grid coordinate transformation of rotation parts with arbitrary rotation centers and rotation axis directions. Combined with the FW-H equation and PO method, GRM can be helpful in the calculation of the dynamic acoustic and RCS characteristics of coaxial tilt-rotor aircraft.
(2) The carpet diagram developed in this paper can intuitively illustrate the influence of the compass angles on the RCS and noise SPL of coaxial tilt-rotor aircraft. The proper compass angles of the two coaxial rotors can be selected by observing the distribution of color bars on the carpet diagrams. Moreover, the scissors angle difference of the two coaxial rotors can have an influence on the distribution regularities of RCS and SPL values on carpet diagrams.
(3) As for the acoustic noise generated by coaxial tilt-rotor aircraft, the variations in the scissors angle difference have influenced the distribution of SPL values on the carpet diagrams. Moreover, the SPL values in fixed-wing mode at an azimuth angle = 180° are obviously lower than those at an azimuth angle = 0° and 90°.
(4) As for the average RCS of coaxial tilt-rotor aircraft, the variations in the scissors angle difference bring complex influences to the distribution of average RCS values on the carpet diagrams. The distribution of average RCS values appears to have clear lines of low values at an azimuth angle = 0°, almost the same value at an azimuth angle = 90°, and a periodic but rather vague distribution at an azimuth angle = 180°. Moreover, the average RCS in helicopter mode is lower than that in fixed-wing mode at an azimuth angle = 90° but higher at an azimuth angle = 0° and 180°.
(5) The stealth design with PPC1 can contribute most to RCS reduction, the stealth design with PPC2 can contribute most to noise reduction at the forehead and rear, and the stealth design with PPC3 can contribute most to noise reduction at the side. Optimal design can be selected according to the requirement of stealth based on the threats.

## Figures and Tables

**Table 1:** Sizes of mesh generations of different parts of aircraft model.

Part	Mesh Size/mm
Fuselage	0.5
Tail	0.1
Wings	0.1
Rotor nacelles	0.1
Rotor shafts	0.1
Blades	0.05

**Table 2:** Numerical values of the geometry parameters of the coaxial tilt-rotor aircraft model.

Parameter	Value	Parameter	Value
$R_{r}$	5.2 m	$L_{f}$	17 m
$d_{rotor}$	2 m	$W_{wing}$	12 m
$d_{axis}$	13.6 m	$W_{tail}$	5.3 m

**Table 3:** Various parameters of the 12 simulation cases considered in this paper.

Case	Azimuth Angle/°	Flight Mode	Calculation
1	0	Helicopter	Noise
2	90	Helicopter	Noise
3	180	Helicopter	Noise
4	0	Fixed-wing	Noise
5	90	Fixed-wing	Noise
6	180	Fixed-wing	Noise
7	0	Helicopter	Dynamic RCS
8	90	Helicopter	Dynamic RCS
9	180	Helicopter	Dynamic RCS
10	0	Fixed-wing	Dynamic RCS
11	90	Fixed-wing	Dynamic RCS
12	180	Fixed-wing	Dynamic RCS

**Table 4:** Fixed parameters of 12 simulation cases considered in this paper.

Elevation Angle/°	Distance between Observer and Aircraft/m	Speed in Helicopter Mode/(km/h)	Speed in Fixed-Wing Mode/(km/h)
45	1000	0	500

**Table 5:** Maximum and minimum SPL values at an azimuth angle = 0° in helicopter mode.

Scissors Angle Difference/°	Maximum Value/dB	Minimum Value/dB
0	56.5	46.1
30	53.6	45.2
60	56.5	46.1
90	53.6	45.2

**Table 6:** Maximum and minimum SPL values at an azimuth angle = 90° in helicopter mode.

Scissors Angle Difference/°	Maximum Value/dB	Minimum Value/dB
0	56.4	46.8
30	53.5	49.2
60	56.4	46.8
90	53.5	49.2

**Table 7:** Maximum and minimum SPL values at an azimuth angle = 180° in helicopter mode.

Scissors Angle Difference/°	Maximum Value/dB	Minimum Value/dB
0	56.5	46.3
30	53.6	45.1
60	56.5	46.3
90	53.6	45.1

**Table 8:** Maximum and minimum SPL values at an azimuth angle = 0° in fixed-wing mode.

Scissors Angle Difference/°	Maximum Value/dB	Minimum Value/dB
0	74.0	67.6
30	71.1	65.1
60	74.0	67.6
90	71.1	65.1

**Table 9:** Maximum and minimum SPL values at an azimuth angle = 90° in fixed-wing mode.

Scissors Angle Difference/°	Maximum Value/dB	Minimum Value/dB
0	72.5	64.6
30	69.5	63.9
60	72.5	64.6
90	69.5	63.9

**Table 10:** Maximum and minimum SPL values at an azimuth angle = 180° in fixed-wing mode.

Scissors Angle Difference/°	Maximum Value/dB	Minimum Value/dB
0	68.2	55.4
30	65.3	58.5
60	68.2	55.4
90	65.3	58.5

**Table 11:** Maximum and minimum average RCS values at an azimuth angle = 0° in helicopter mode.

Scissors Angle Difference/°	Maximum Value/dBsm	Minimum Value/dBsm
0	7.8	6.3
30	7.6	6.6
60	7.8	6.3
90	7.6	6.6

**Table 12:** Highest and lowest average RCS values at an azimuth angle = 90° in helicopter mode.

Scissors Angle Difference/°	Maximum Value/dBsm	Minimum Value/dBsm
0	25.6259	25.5916
30	25.6154	25.5873
60	25.6251	25.5898
90	25.6176	25.5849

**Table 13:** Highest and lowest average RCS values at an azimuth angle = 180° in helicopter mode.

Scissors Angle Difference/°	Maximum Value/dBsm	Minimum Value/dBsm
0	5.2	3.9
30	5.1	3.8
60	5.2	4.0
90	5.2	3.9

**Table 14:** Maximum and minimum average RCS values in fixed-wing mode at an azimuth angle = 0°.

Scissors Angle Difference/°	Maximum Value/dBsm	Minimum Value/dBsm
0	5.0	3.2
30	4.5	3.3
60	5.0	3.2
90	4.5	3.3

**Table 15:** Highest and lowest average RCS values at an azimuth angle = 90° in fixed-wing mode.

Scissors Angle Difference/°	Maximum Value/dBsm	Minimum Value/dBsm
0	29.5128	29.4957
30	25.5128	25.4964
60	29.5128	29.4957
90	25.5128	25.4964

**Table 16:** Highest and lowest average RCS values at an azimuth angle = 180° in fixed-wing mode.

Scissors Angle Difference/°	Maximum Value/dBsm	Minimum Value/dBsm
0	3.8	2.0
30	3.4	2.1
60	3.8	2.0
90	3.4	2.1

**Table 17:** Noise SPL and average RCS values with CSM phase parameters of Equations (43)–(46).

	Helicopter Mode		Fixed-Wing Mode	
	Noise SPL/dB	Average RCS/dBsm	Noise SPL/dB	Average RCS/dBsm
Azimuth angle = 0°	49.1	6.7	69.5	3.4
Azimuth angle = 90°	49.1	25.6	64.9	29.5
Azimuth angle = 180°	49.1	4.0	58.1	2.8

**Table 18:** Noise SPL and average RCS values with CSM phase parameters of Equations (47)–(50).

	Helicopter Mode		Fixed-Wing Mode	
	Noise SPL/dB	Average RCS/dBsm	Noise SPL/dB	Average RCS/dBsm
Azimuth angle = 0°	45.1	6.7	65.1	4.0
Azimuth angle = 90°	53.5	25.6	68.9	29.5
Azimuth angle = 180°	45.1	4.7	58.6	2.8

**Table 19:** Noise SPL and average RCS values with CSM phase parameters of Equations (51)–(54).

	Helicopter Mode		Fixed-Wing Mode	
	Noise SPL/dB	Average RCS/dBsm	Noise SPL/dB	Average RCS/dBsm
Azimuth angle = 0°	53.4	7.2	71.0	4.1
Azimuth angle = 90°	41.3	25.6	64.2	29.5
Azimuth angle = 180°	53.4	4.7	65.3	2.8

**Table 20:** Noise SPL and average RCS values with PPC1.

	Helicopter Mode		Fixed-Wing Mode	
	Noise SPL/dB	Average RCS/dBsm	Noise SPL/dB	Average RCS/dBsm
Azimuth angle = 0°	49.1	6.7	69.5	3.4
Azimuth angle = 90°	49.1	25.6	64.9	29.5
Azimuth angle = 180°	49.1	4.0	58.1	2.8

**Table 21:** Noise SPL and average RCS values with PPC2.

	Helicopter Mode		Fixed-Wing Mode	
	Noise SPL/dB	Average RCS/dBsm	Noise SPL/dB	Average RCS/dBsm
Azimuth angle = 0°	45.1	6.7	65.1	4.0
Azimuth angle = 90°	53.5	25.6	68.9	29.5
Azimuth angle = 180°	45.1	4.5	58.6	2.8

**Table 22:** Noise SPL and average RCS values with PPC3.

	Helicopter Mode		Fixed-Wing Mode	
	Noise SPL/dB	Average RCS/dBsm	Noise SPL/dB	Average RCS/dBsm
Azimuth angle = 0°	53.4	7.2	71.0	4.1
Azimuth angle = 90°	41.3	25.6	64.2	29.5
Azimuth angle = 180°	53.4	4.7	65.3	2.8

**Table 23:** Reduction values of noise SPL and average RCS of PPC1, PPC2 and PPC3.

	Helicopter Mode		Fixed-Wing Mode	
	Noise Reduction/dB	RCS Reduction/dBsm	Noise Reduction/dB	RCS Reduction/dBsm
Azimuth angle = 0°	PPC1: 7.4	PPC1: 1.1	PPC1: 4.5	PPC1: 1.6
	PPC2: 11.4	PPC2: 1.1	PPC2: 8.9	PPC2: 1
	PPC3: 3.1	PPC3: 0.6	PPC3: 3	PPC3: 0.9
Azimuth angle = 90°	PPC1: 7.3	PPC1: 0	PPC1: 7.6	PPC1: 0
	PPC2: 2.9	PPC2: 0	PPC2: 3.6	PPC2: 0
	PPC3: 15.1	PPC3: 0	PPC3: 8.3	PPC3: 0
Azimuth angle = 180°	PPC1: 7.4	PPC1: 1.2	PPC1: 10.1	PPC1: 1
	PPC2: 11.4	PPC2: 0.7	PPC2: 9.6	PPC2: 1
	PPC3: 3.1	PPC3: 0.5	PPC3: 2.9	PPC3: 1
